# Dosage and Cell Line Dependent Inhibitory Effect of bFGF Supplement in Human Pluripotent Stem Cell Culture on Inactivated Human Mesenchymal Stem Cells

**DOI:** 10.1371/journal.pone.0086031

**Published:** 2014-01-17

**Authors:** Tara Quang, Maribel Marquez, Giselle Blanco, Yuanxiang Zhao

**Affiliations:** Biological Sciences Department, California State Polytechnic University at Pomona, Pomona, California, United States of America; Baylor College of Medicine, United States of America

## Abstract

Many different culture systems have been developed for expanding human pluripotent stem cells (hESCs and hiPSCs). In general, 4–10 ng/ml of bFGF is supplemented in culture media in feeder-dependent systems regardless of feeder cell types, whereas in feeder-free systems, up to 100 ng/ml of bFGF is required for maintaining long-term culture on various substrates. The amount of bFGF required in native hESCs growth niche is unclear. Here we report using inactivated adipose-derived human mesenchymal stem cells as feeder cells to examine long-term parallel cultures of two hESCs lines (H1 and H9) and one hiPSCs line (DF19-9-7T) in media supplemented with 0, 0.4 or 4 ng/ml of bFGF for up to 23 passages, as well as parallel cultures of H9 and DF19 in media supplemented with 4, 20 or 100 ng/ml bFGF for up to 13 passages for comparison. Across all cell lines tested, bFGF supplement demonstrated inhibitory effect over growth expansion, single cell colonization and recovery from freezing in a dosage dependent manner. In addition, bFGF exerted differential effects on different cell lines, inducing H1 and DF19 differentiation at 4 ng/ml or higher, while permitting long-term culture of H9 at the same concentrations with no apparent dosage effect. Pluripotency was confirmed for all cell lines cultured in 0, 0.4 or 4 ng/ml bFGF excluding H1-4 ng, as well as H9 cultured in 4, 20 and 100 ng/ml bFGF. However, DF19 demonstrated similar karyotypic abnormality in both 0 and 4 ng/ml bFGF media while H1 and H9 were karyotypically normal in 0 ng/ml bFGF after long-term culture. Our results indicate that exogenous bFGF exerts dosage and cell line dependent effect on human pluripotent stem cells cultured on mesenchymal stem cells, and implies optimal use of bFGF in hESCs/hiPSCs culture should be based on specific cell line and its culture system.

## Introduction

Since the inception of human embryonic stem cell (hESCs) culture in 1998 and subsequently human induced pluripotent stem cell (hiPSCs) in 2007, these cells have been universally cultured in various artificial systems supplemented with exogenous bFGF, a growth factor that is considered critical for maintaining pluripotency and preventing differentiation through autocrine and paracrine bFGF signaling [1], [2]. Since the initial culture of hESCs on mouse embryonic fibroblast (MEF) feeder cells in media supplemented with knock out serum replacement (KOSR) and 4 ng/ml bFGF [3], a number of new culture systems have been developed to eliminate use of animal feeder cells, which can be categorized into human feeder cell-dependent system and feeder cell-free system. Several human cell types have been successfully used to establish and sustain hESCs culture, including human foreskin fibroblasts (HFF) [4], [5], [6], [7], fetal skin fibroblasts [8], [9], [10], fetal muscle fibroblasts [8], [10], fetal lung fibroblasts [11], [12], embryonic fibroblasts [13], [14], [15], adult skin fibroblasts [8], [16], adult placental fibroblasts (HPF) [17], [18], and adult fallopian tube epithelial cells [10]. All of the above human feeder cell based cultures used 4–10 ng/ml of bFGF in conjunction with either 15–20% KOSR or human serum, along with other shared components (1% non-essential amino acids, 1–4 mM glutamine and 0.1 mM b-mercaptoethanol) diluted in selected basal medium. An alternative chemically defined RegES medium (with 8 ng/ml bFGF and others) was also shown to support hESCs long-term self-renewal on human foreskin fibroblasts [19].

One of the limiting factors for using human feeder cells to culture hESCs is the presence of unknown factors supplied by the feeder cells that might play a role in supporting the growth of hESCs. Hence, many feeder-free culture systems have been developed that combine chemically defined media in conjunction with different types of cell-attachment substrates [20], [21], [22], [23], [24], [25], [26]. Several chemically defined medium formulas have been commercialized, including HESCO medium (160 ng/ml insulin, 100 ng/ml Wnt3a, 100 ng/ml BAFF, 88 ng/ml transferrin, 4 ng/ml bFGF and others) that supports hESCs culture on both matrigel and fibronectin [24], StemPro medium (200 ng/ml LR^3^-IGF1, 10 ng/ml transferring, 10 ng/ml HRG1β, 10 ng/ml Activin A, 8 ng/ml FGF2 and others) that supports hESCs culture on matrigel [25], and TeSR1 medium (100 µg/ml Insulin, 0.3 ng/ml TGFβ, 55 µg/ml Transferrin, 50 µg/ml GABA, 200 ng/ml Pipecolic Acid, 50 ng/ml bFGF and others) that also supports hESCs culture on matrigel [26]. More recently, the mTeSR1 media was further simplified to E8 medium (20 µg/ml Insulin, 2 ng/ml TGFβ, 11 µg/ml Transferrin, 100 ng/ml bFGF and others) [27].

Similar to hESCs, both feeder cell-dependent and feeder cell-free systems supplemented with exogenous bFGF have been applied to culture hiPSCs. Following the footsteps of two pioneering studies on generating hiPSCs lines [28], [29], hiPSCs are conventionally maintained on MEF feeder cells with 20% KOSR supplemented with a wide range of bFGF, 4–100 ng/ml [30], [31]. A number of studies have also described the successful culture of hiPSCs on human adult feeder cells supplemented with 4–20 ng/ml bFGF [19], [32], [33], [34], [35], [36], [37]. In addition, several feeder-free systems applicable for hESCs culture have also been shown to support hiPSCs culture, including TeSR1 or E8 medium on matrigel, vitronectin, synthetic peptide substrate or laminin [27], [38], [39], [40], [41], StemPro hESC SFM on Geltrix [37], and FTDA medium (0.5 ng/ml TGFβ1, 5 ng/ml Activin A, 50 nM Dorsomorphin, 10 ng/ml bFGF and others) on matrigel [41].

In summary, of the numerous culture conditions developed for hESCs/hiPSCs, in general 4–10 ng/ml of exogenous bFGF is supplemented when they are cultured in human feeder cell-dependent systems, whereas in feeder-free systems, a much higher concentration of bFGF or other growth factors is required. It is interesting to note that these two culture systems are not interchangeable, as TeSR1 medium could not maintain hESCs culture on HFF feeder cells, nor could HESCO medium [19], [42]. This implies potential incompatibility between the human feeder cell niche and high concentrations of exogenous bFGF supplement for supporting hESCs/hiPSCs growth. Indeed, against the conventional wisdom, one group recently reported that two hESCs lines, H1 and HSF6, could be maintained on 3 types of human feeder cells including HFF, HPF and bone marrow stromal cells without the need of exogenous bFGF supplement [43], [44]. Subsequently, it was shown that two other hESCs lines, H9 and AND1, could be long-term sustained in conditioned media by human mensenchymal stem cells, but not HFF, in the absence of exogenous bFGF [45]. However, in order to better understand the effect of exogenous bFGF on the long-term culture of both hESCs and hiPSCs on human feeder cells, systematic comparison of different cell lines cultured with or without bFGF supplement is required. Here we report using inactivated Ad-hMSCs as feeder cells to support the culture of both hESCs and hiPSCs and examined parallel cultures of two hESCs lines (H1 and H9) and one hiPSCs line (DF19-9-7T) in media supplemented with 0, 0.4 or 4 ng/ml of bFGF for up to 23 passages, as well as parallel cultures of H9 and DF19 in media supplemented with 4, 20 or 100 ng/ml bFGF for up to 13 passages for systematic comparison. Our results for the first time demonstrated dosage dependent inhibitory effect of bFGF supplement on growth expansion, clonogenicity and thawing efficiency across all three cell lines cultured on hMSCs feeder cells, as well as cell line dependent effect of bFGF on growth, differentiation and chromosomal stability.

## Results

### Long-term culture of H1, H9 or DF19 in media supplemented with 0, 0.4 or 4 ng/ml of bFGF

It is not clear whether any significant differences existed between hESCs/hiPSCs cultures supplemented with and without bFGF on human feeder cells. To investigate this, two hESCs lines (H1, P32 and H9, P25) and one hiPSCs line (DF19) were cultured in parallel in hESCs/hiPSCs media (80% DMEM/F12, 20% KOSR, 1% NEAA, 1 mM Glutamine and 0.07 µM beta-mercaptoethanol) supplemented with 0, 0.4 or 4 ng/ml of bFGF on Ad-hMSCs inactivated by mitomycin C treatment (MtC-hMSCs). Both H1 and H9 were originally received and expanded on MEF feeder cells supplemented with 4 ng/ml bFGF, from which colonies were evenly split by collagenase onto 3 wells of MtC-hMSC feeder cells supplemented with 0, 0.4 or 4 ng/ml exogenous bFGF. Subsequent cultures were designated as H1-0 ng, H1-0.4 ng or H1-4 ng at P32+n or H9-0 ng, H9-0.4 ng or H9-4 ng at P25+n, respectively, where n indicates the number of passages cultured on MtC-hMSCs feeder cells. DF19 was originally received and expanded in mTeSR1 on matrigel before it was transitioned onto MtC-hMSCs feeder cells in a similar fashion and were designated as DF19-0 ng, DF19-0.4 ng or DF19-4 ng at P29+n. All three cultures were tested negative for mycoplasma contamination after long-term culture in 0 ng/ml bFGF (Figure S1).

**Figure 1:**
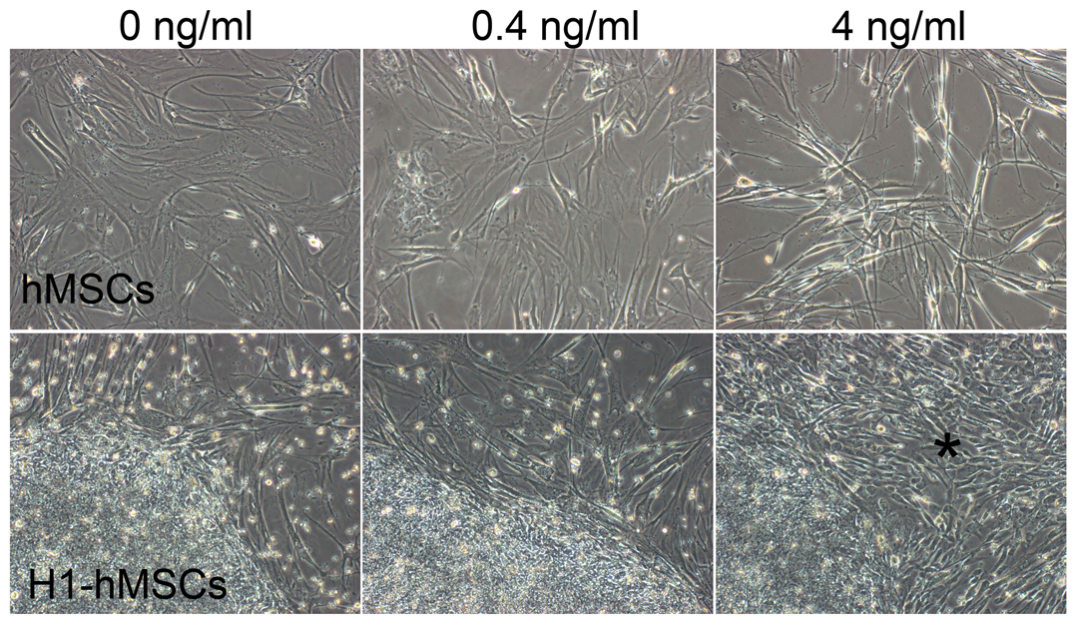
Morphological changes of hMSCs feeder cells and H1 cells in media supplemented with 0, 0.4 or 4/ml bFGF. Upper row: hMSCs feeder cells presented distinct morphologies in media supplemented with 0, 0.4 or 4 ng/ml bFGF. Bottom row: H1-4 ng culture differentiated into short spindle-shaped fibroblast like cells at P32+8 as indicated by the asterisk (*). See also Figure S1.

Morphologically hMSCs feeder cells appeared distinct when exposed to the hESCs/hiPSCs media supplemented with different concentrations of bFGF, appearing flat and spread-out in media with 0 ng/ml bFGF, elongated and string-shaped with 4 ng/ml bFGF, and somewhat in between with 0.4 ng/ml bFGF (top row, Figure 1). All treatment groups of each cell line were split identically initially. After a few passages however, it was obvious that colonies in 4 ng/ml bFGF media had a slower growth rate than those in 0 and 0.4 ng/ml bFGF media for all three cell lines and hence had to be split at a lower ratio in the subsequent passages, in order to keep up with the same splitting frequency as the other two conditions. The difference was more notable for H1 and DF19 cells. No significant growth difference was visually noticed between 0 vs. 0.4 ng/ml wells during the long-term culture.

All cultures were continued for up to 23 passages, except for H1 cultured with 4 ng/ml bFGF (H1-4 ng), which differentiated extensively at P32+8, resulting in numerous short spindle-shaped fibroblast-like cells (* in bottom row, Figure 1). The same dramatic onset of differentiation was observed repeatedly at P32+8 when frozen culture from earlier passages was thawed out in attempt to reinstate long-term culture. Continued culture beyond P32+8 resulted in more aggregated differentiation, and the culture was ceased at P32+10.

### H1, H9 and DF19 had slower growth rate in media supplemented with 4 ng/ml of bFGF vs. 0 ng/ml bFGF

**Figure 2:**
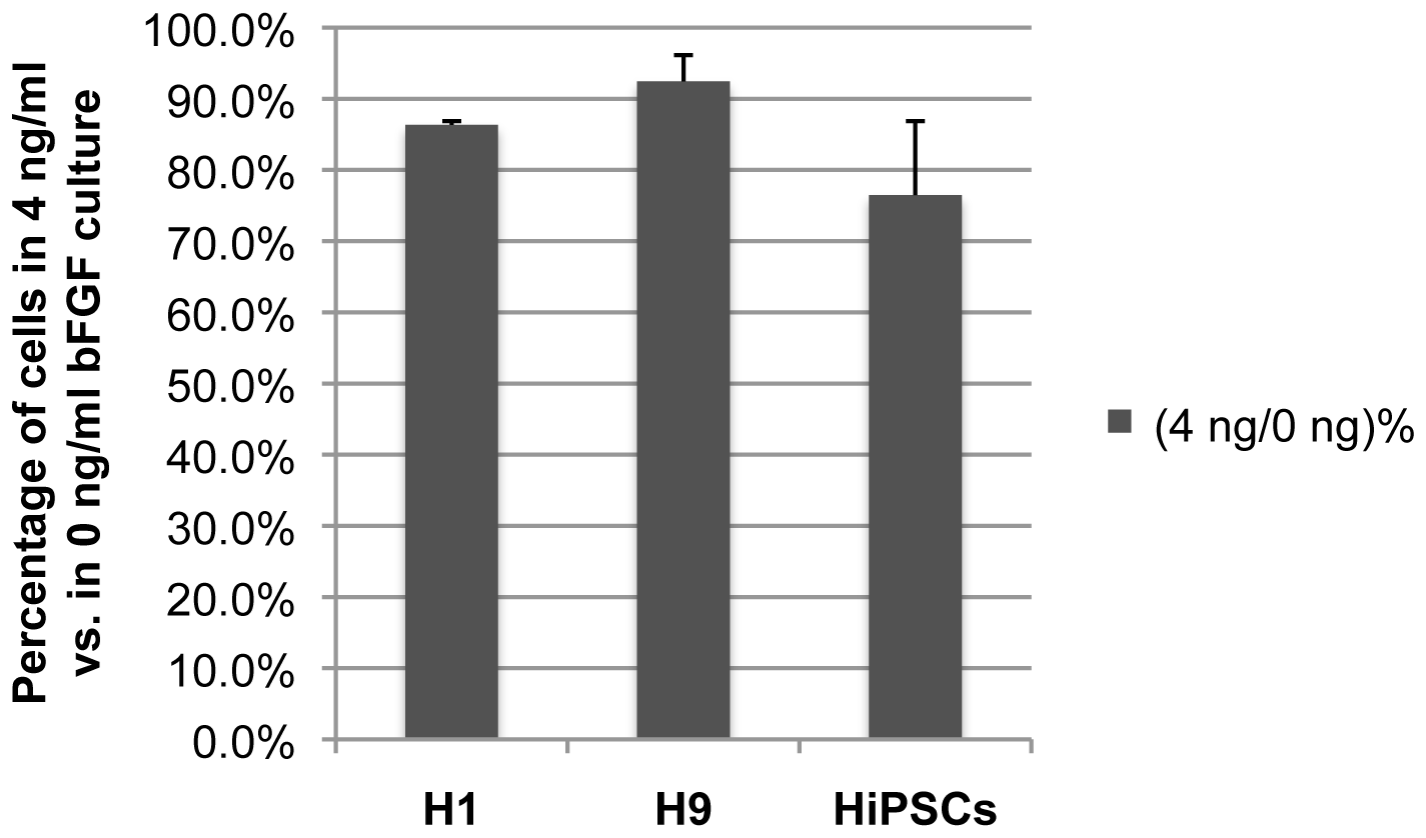
H1, H9 and DF19 had higher growth rate in media supplemented with 0/ml vs. 4 ng/ml bFGF. Cells that have been cultured with 0 or 0.4/ml bFGF supplement were split evenly into media with 0 or 4 ng/ml bFGF, 3 wells of each, and subsequently counted after 8 days of culture. The average percentage of cells in 4 ng/ml culture vs. 0 ng/ml was presented for each cell line (n = 3). Error bars represent standard deviation among triplicate repeats.

**Figure 3:**
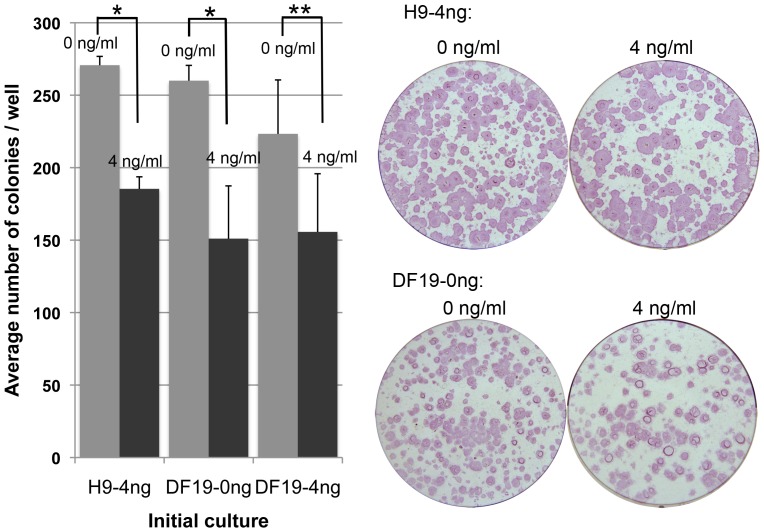
Culture with 0/ml bFGF had increased number of colonies compared to culture with 4 ng/ml bFGF. H9-4 ng, DF19-0 ng or DF19-4 ng culture was each split evenly 1∶6 into media supplemented with 0 or 4 ng/ml bFGF, 3 wells of each. Colonies were imaged and counted after 8 days of culture. The average number of colonies from triplicate wells was presented for each culture (n = 1). Error bars represent standard deviation among triplicates. Corresponding representative images were taken after ALP staining. *: P<0.01; **: P<0.1.

To quantify the visual observation of differential growth rates between 0 and 4 ng/ml cultures for each cell line, cells that have been cultured with 0 or 0.4 ng/ml bFGF supplement for 3 or more passages were split evenly 1∶6 by collagenase and plated into 0 vs. 4 ng/ml bFGF media, 3 wells of each. After 8 days of growth, colonies in each well were dissociated by accutase into single cells and counted. The same assay was repeated three times for each cell line and the 0 ng/ml cultures consistently yielded higher number of total cells compared to the 4 ng/ml cultures across all three cell lines (Figure 2). Similar results were observed when the same assay was repeated with H9 and DF19 cells that were initially cultured with 4 ng/ml bFGF (H9-4 ng/DF19-4 ng) and subsequently split into 0 vs. 4 ng/ml culture, with the number of colonies in 4 ng/ml bFGF culture vs. in 0 ng/ml bFGF culture ((4 ng/0 ng)%) as 75% and 91%, respectively. To investigate whether the increased cell number in 0 ng/ml culture was a result of increased colony numbers or size, H9-4 ng, DF19-0 ng and DF19-4 ng cultures were each chosen as initial cultures and evenly split 1∶6 into 0 vs. 4 ng/ml bFGF media, 3 wells of each. After 8 days of culture, colonies were fixed, ALP stained, imaged and counted. In both cases with H9-4 ng and DF19-0 ng as initial cultures, there was significantly higher number of colonies in 0 ng/ml culture wells compared to 4 ng/ml wells (Figure 3, *P<0.01). The difference was less significant when DF19-4 ng was the initial culture (Figure 3, **P<0.1). This indicates that the observed higher growth rate in 0 ng/ml cultures vs. 4 ng/ml cultures was at least partially attributable to increased colony numbers.

### Exogenous bFGF inhibits clonogenicity across all three cell lines

**Figure 4:**
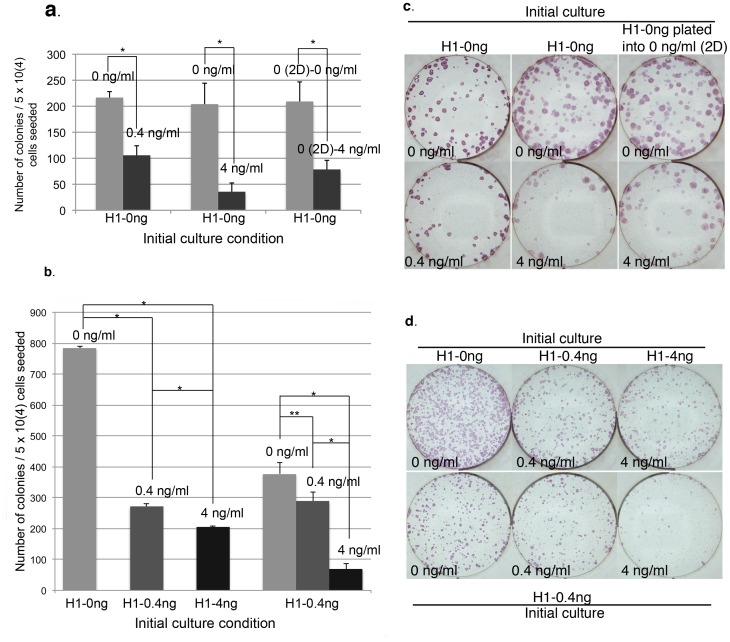
H1 clonogenicity is inhibited by exogenous bFGF in a dosage dependent manner. (a). Initial H1-0 ng culture was plated as single cells into 0 vs. 0.4 ng/ml bFGF, 0 vs. 4 ng/ml bFGF, or 0 vs. 4 ng/ml bFGF after 2 days (2D) of culture in 0 ng/ml bFGF media for clonogenicity comparison. (b). Representative images of ALP stained cultures as described in (a). (c). Left-hand three columns: Initial H1-0 ng, H1-0.4 ng and H1-4 ng culture were each plated as single cells into its corresponding culture condition for clonogenicity comparison. Right-hand three columns: Initial H1-0.4 ng culture was plated as single cells into 0, 0.4 and 4 ng/ml bFGF media for clonogenicity comparison. (d). Representative images of ALP stained cultures as described in (c), with top row images corresponding to the left-hand three columns in 4c and bottom row images corresponding to the right-hand three columns in 4c. The average number of colonies from triplicate wells was presented for each culture (n = 1) except for the right-hand three columns in 4c, which represent the average of duplicate wells (n = 2). Error bars represent standard deviation. *: P<0.01.

The increased number of colonies in 0 ng/ml vs. 4 ng/ml bFGF cultures suggests that exogenous bFGF might exert an inhibitory effect on pluripotent stem cell's clonogenicity. To assess this, single cell colonization assay was carried out for all three cell lines. H1-0 ng at P32+4 was dissociated by accutase into single cell solution and plated at 5×10^4^ cells/well directly into media supplemented with 0 vs. 0.4 ng/ml bFGF, or 0 vs. 4 ng/ml bFGF media, 3 wells of each in each group. Colonies were fixed, ALP stained, imaged and counted after 14 days of culture. In both cases, the number of colonies formed in 0.4 or 4 ng/ml bFGF media were significantly lower than those in 0 ng/ml bFGF media (Figures 4a and 4b). To examine whether the increased clonogenicity in 0 ng/ml bFGF media was due to enhanced single cell attachment during initial plating, the same H1-0 ng single cells at P32+4 were also plated in 0 ng/ml bFGF media for 48 hours before switching to 0 (0 (2D)-0 ng/ml) vs. 4 ng/ml (0 (2D)-4 ng/ml) bFGF media, 3 wells of each. Despite an increase in the number of colonies recovered in 4 ng/ml bFGF media as a result of the pre-condition in 0 ng/ml media, it is still significantly lower than those in 0 ng/ml bFGF media (Figures 4a and 4b), indicating that exogenous bFGF had an inhibitory effect on both the initial single cell attachment as well as its long-term survival. To eliminate potential bias as a result of adaptation in 0 ng/ml bFGF media for the initial H1-0 ng cells used in the above assay, H1-0 ng, H1-0.4 ng and H1-4 ng culture at P32+6 were each dissociated into single cells and plated directly into its corresponding initial culture condition at 5×10^4^ cells/well, 3 wells of each. Again, the H1-0.4 ng and H1-4 ng cultures had significantly lower number of colonies than the H1-0 ng culture (Figures 4c and 4d). In addition, the number of colonies in the H1-0.4 ng culture was significantly greater than those in the H1-4 ng culture as well (Figure 4c). Finally, we also tested in parallel single cell plating of initial H1-0.4 ng culture at P32+6 into 0, 0.4 and 4 ng/ml bFGF media at 5×10^4^ cells/well, 2 wells of each on the same 6-well plate. The same experiment was repeated once and in both cases, there was significantly greater number of colonies in the 0 or 0.4 ng/ml bFGF culture compared to those in the 4 ng/ml bFGF culture (Figures 4c and 4d). It is noteworthy that the difference between 0 vs. 0.4 ng/ml bFGF culture in this case appeared to be relatively reduced compared to the difference observed when the initial culture was H1-0 ng as shown in figure 4a, implying that pre-adaptation to a specific bFGF concentration could help enhance the survival rate of H1 single cells in the same condition, though it could not overcome the overall inhibitory effect of exogenous bFGF. In summary, the above results indicate that H1 cell's clonogenicity rate is inhibited by exogenous bFGF in a dosage dependent manner when using inactivated hMSCs as feeder cells.

**Figure 5:**
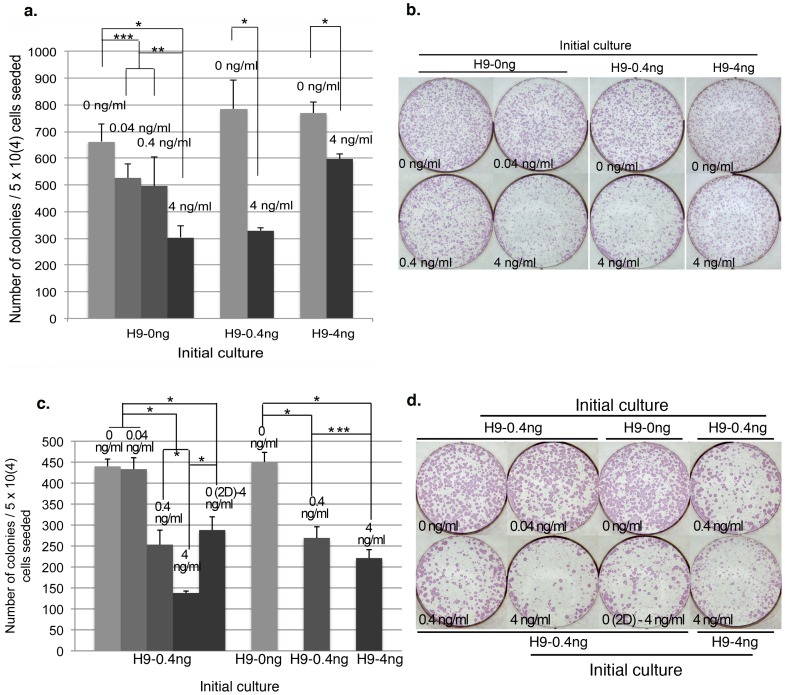
H9 clonogenicity is inhibited by exogenous bFGF in a dosage dependent manner. (a). Initial H9-0 ng or H9-0.4 ng/H9-4 ng culture was dissociated into single cells and plated into 0 vs. 0.04 vs. 0.4 vs. 4 ng/ml bFGF media, or 0 vs. 4 ng/ml bFGF media, respectively, at 5×10^4^ cells/well. (b). Representative images of ALP stained cultures as described in (a). (c). Left-hand five columns: Initial H9-0.4 ng culture was dissociated into single cells and plated into 0 vs. 0.04 vs. 0.4 vs. 4 ng/ml bFGF or 4 ng/ml bFGF after 2 days (2D) of culture in 0 ng/ml bFGF media. Right-hand three columns: initial H9-0 ng, H9-0.4 ng and H9-4 ng culture were each plated as single cells into its corresponding culture condition, at 5×10^4^ cells/well. (d). Representative images of ALP stained cultures as described in (c). The average number of colonies from triplicate wells was presented for each culture (n = 1). Error bars represent standard deviation. *: P<0.01; **: P<0.05; ***: P<0.1.

Similar single cell colonization assays were carried out for H9 and DF19. For H9, initial H9-0 ng at P25+11 was dissociated by accutase into single cells and plated into 0, 0.04, 0.4 or 4 ng/ml bFGF media, at 5×10^4^ cells/well, 3 wells of each. Consistent with the results in H1 cells, exogenous bFGF also inhibited H9 clonogenicity in a dosage dependent manner, though the overall inhibition appeared less potent as compared to in H1, with (4 ng/0 ng%) being roughly about 45% (Figure 5a) in H9 vs. 25% in H1 (Figures 4a–c), indicating that H9 might be less sensitive to exogenous bFGF compared to H1. Additionally when initial H9-0.4 ng or H9-4 ng culture was dissociated and plated into 0 vs. 4 ng/ml bFGF media, similar to what was observed with H1 cells, the difference between 0 vs. 4 ng/ml bFGF culture was relatively reduced when the initial culture was H9-4 ng as compared to H9-0.4 ng or H9-0 ng (Figure 5a), indicating that pre-adaptation to a specific bFGF concentration could help enhance the survival rate of H9 single cells in the same condition as well. To eliminate potential bias as a result of adaptation in 0 ng/ml bFGF media for the initial H9-0 ng cells used in the above assay, H9-0 ng, H9-0.4 ng and H9-4 ng initial culture were each dissociated into single cells and plated directly into its corresponding initial culture condition at 5×10^4^ cells/well, 3 wells of each. Again, the H9-0.4 ng and H9-4 ng cultures had significantly lower number of colonies than the H9-0 ng culture (Figures 5c and 5d). In addition, we also tested single cell plating of initial H9-0.4 ng culture into 0, 0.04, 0.4 and 4 ng/ml bFGF media at 5×10^4^ cells/well, 3 wells of each. Consistently there was significantly greater number of colonies in the 0 or 0.04 ng/ml bFGF culture compared to those in the 0.4 or 4 ng/ml bFGF culture (Figures 5c and 5d). Similar to H1 culture, pre-condition of the same H9-0.4 ng single cells in 0 ng/ml bFGF media for 2 days before switching to 4 ng/ml bFGF media (0 (2D)-4 ng/ml) significantly enhanced the total number of colonies recovered compared to those directly seeded in 4 ng/ml bFGF media but not sufficient to rescue to the same level as those plated into 0 or 0.04 ng/ml bFGF media (Figure 5c and 5d), indicating that exogenous bFGF had an inhibitory effect on both the initial single cell attachment as well as its long-term survival.

**Figure 6:**
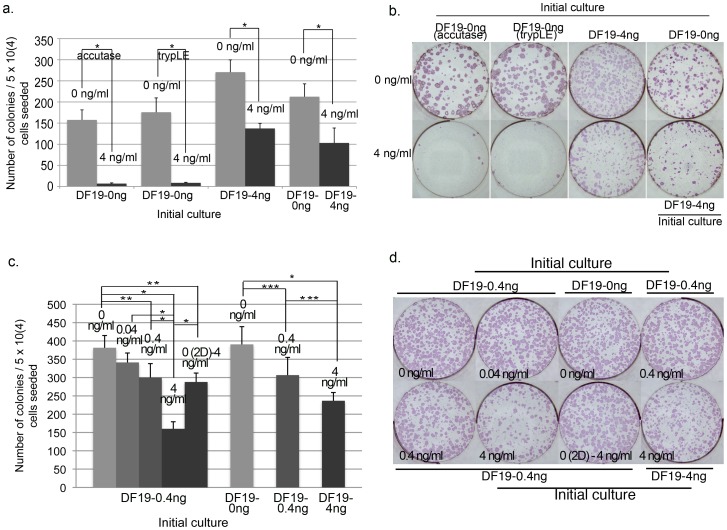
DF19 clonogenicity is inhibited by exogenous bFGF in a dosage dependent manner. (a) Initial DF19-0 ng was dissociated into single cells by accutase or trypLE and plated into 0 vs. 4 ng/ml bFGF media at 5×10^4^ cells/well. The same experiment was carried out with initial DF19-4 ng cells using accutase. In addition, DF19-0 ng and DF19-4 ng were also plated as single cells into its corresponding 0 or 4 ng/ml bFGF media respectively. (b). Representative images of ALP stained cultures as described in (a). (c). Left-hand five columns: Initial DF19-0.4 ng culture was dissociated into single cells and plated into 0 vs. 0.04 vs. 0.4 vs. 4 ng/ml bFGF or 4 ng/ml bFGF after 2 days (2D) of culture in 0 ng/ml bFGF media. Right-hand three columns: initial DF19-0 ng, DF19-0.4 ng and DF19-4 ng culture were each plated as single cells into its corresponding culture condition, at 5×10^4^ cells/well. (d). Representative images of ALP stained cultures as described in (c). The average number of colonies from triplicate wells was presented for each culture (n = 1). Error bars represent standard deviation. *: P<0.01; **: P = <0.05; ***: P = <0.1.

For DF19, initial DF19-0 ng or DF19-4 ng culture was dissociated by accutase or TrypLE into single cells and plated into 0 vs. 4 ng/ml bFGF media at 5×10^4^ cells/well, 3 wells of each. In addition, the same initial cells were also plated as single cells into its corresponding initial culture media respectively, 3 wells of each. Similar to results observed with both H1 and H9 cells, DF19 clonogenicity was also inhibited by bFGF regardless of the dissociation enzyme used (Figures 6a and 6b). Additionally, the difference between 0 vs. 4 ng/ml bFGF culture was relatively reduced when the initial culture was DF19-4 ng as compared to DF19-0 ng (Figure 6a), indicating that pre-adaptation to a specific bFGF concentration could help enhance the survival rate of DF19 single cells in the same condition, similar to both H1 and H9 cells described above. In addition, DF19-0 ng, DF19-0.4 ng and DF19-4 ng initial culture were each dissociated into single cells and plated directly into its corresponding initial culture condition at 5×10^4^ cells/well, 3 wells of each. Again, the number of colonies decreased as the bFGF concentration increased, with significantly lower number of colonies in the DF19-4 ng culture than the DF19-0 ng culture (Figures 6c and 6d). Finally, we also tested single cell plating of initial DF19-0.4 ng culture into 0, 0.04, 0.4 and 4 ng/ml bFGF media at 5×10^4^ cells/well, 3 wells of each. Consistently there was significantly greater number of colonies in the 0, 0.04 or 0.4 ng/ml bFGF culture compared to those in the 4 ng/ml bFGF culture (Figures 6c and 6d). Similar to H1 and H9 cultures, pre-condition of the same DF19-0.4 ng single cells in 0 ng/ml bFGF media for 2 days before switching to 4 ng/ml bFGF media (0 (2D)-4 ng/ml) significantly enhanced the total number of colonies recovered compared to those directly seeded in 4 ng/ml bFGF media, but not sufficient to rescue to the same level as those plated into 0 ng/ml bFGF media (Figure 6c and 6d), indicating that exogenous bFGF had an inhibitory effect on both the initial single cell attachment as well as its long-term survival.

**Figure 7:**
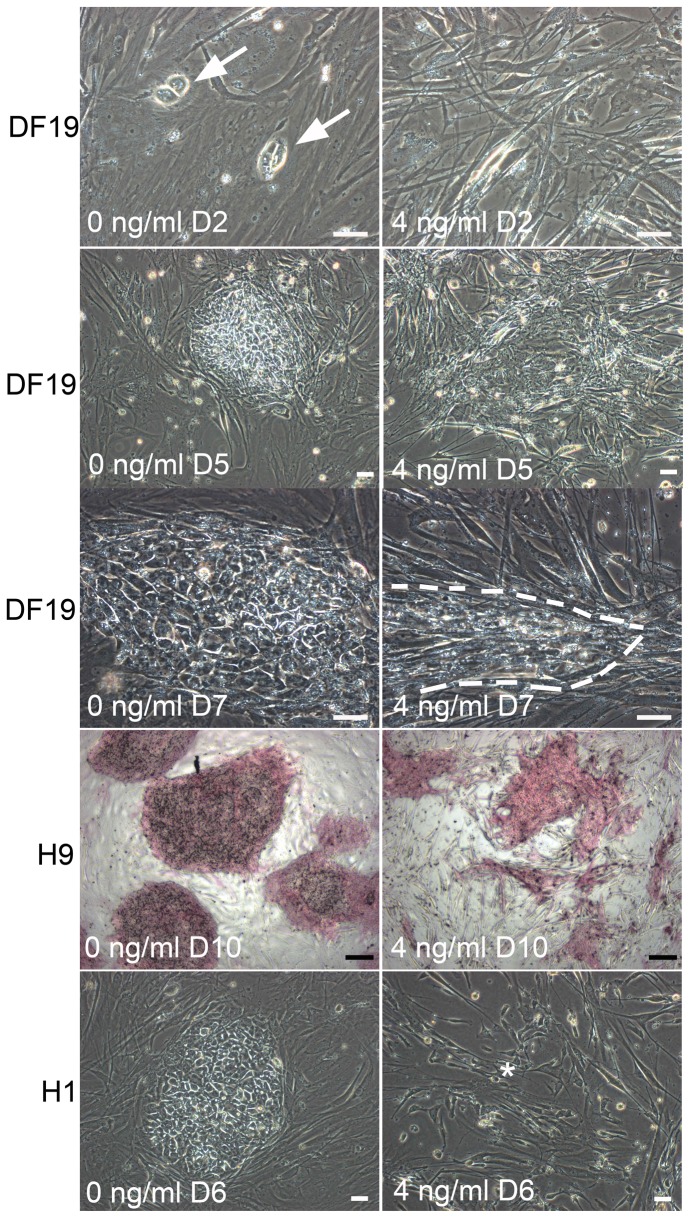
Morphological differences in single cell cultures with 0 vs. 4/ml bFGF. Top row: dividing cells were observed in 0 ng/ml bFGF culture but not in 4 ng/ml bFGF culture; Second row: degenerative colonies were observed in 4 ng/ml bFGF culture but not in 0 ng/ml culture; Third and fourth rows: Surviving colonies in 4 ng/ml bFGF culture were smaller and slender than in 0 ng/ml culture; Bottom row: Fibroblast-like differentiated cells (*) were observed in 4 ng/ml bFGF culture but not in 0 ng/ml culture, both of which were derived from H1-4 ng at P31+6. Scales in white: 50 µM; Scales in black: 200 µM.

Morphologically, after 2 days of plating as single cells, dividing pluripotent stem cells in 0 ng/ml cultures were readily visible under microscope for all three cell lines, whereas such cells were much more scarce in 4 ng/ml bFGF cultures (Figure 7, top row). As time passes, degenerating colonies started to appear in 4 ng/ml bFGF cultures (Figure 7, second row) and surviving colonies typically appeared smaller and slender than those in 0 ng/ml bFGF cultures, particularly with H9 and DF19 cells (Figure 7, third and fourth rows). The above observations indicated that exogenous bFGF was detrimental to both single cell attachment and survival. Finally, similar to those observed in H1-4 ng long-term culture, single cell culture in 4 ng/ml bFGF media that was derived from initial H1-4 ng culture at P31+6 also had spontaneous fibroblast-like differentiation (Figure 7, asterisk in bottom row), which was not observed in parallel cultures in 0 or 0.4 ng/ml bFGF, nor in H9 or DF19 culture.

### Exogenous bFGF promotes colony growth and inhibits differentiation of pluripotent stem cells cultured on MEF feeder cells

**Figure 8:**
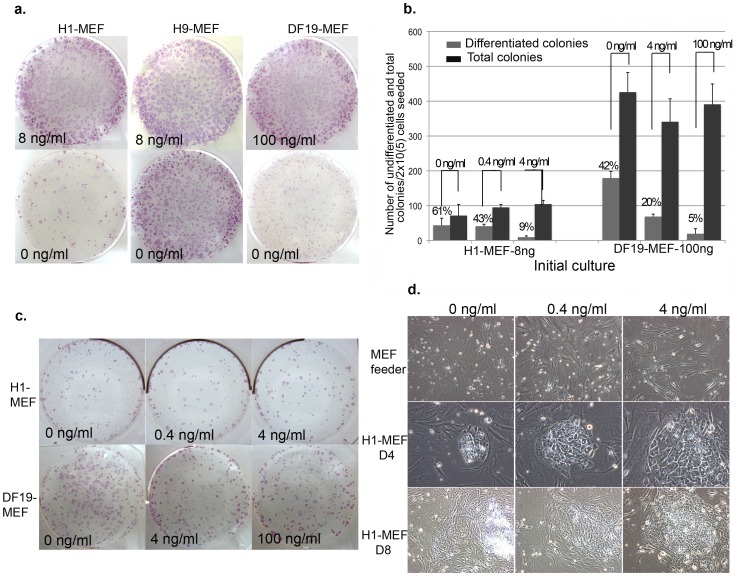
Exogenous bFGF promotes colony growth and inhibits differentiation of pluripotent stem cells cultured on MEF. (a). ALP stained images of H1-MEF, H9-MEF and DF19-MEF cultured in 0 vs. 8 or 0 vs. 100 ng/ml bFGF media for 12 days. (b). H1-MEF-8 ng (P32) and DF19-MEF-100 ng (P33) single cells were plated at 2×10^5^ cells/well into 0, 0.4 and 4 ng/ml bFGF media or 0, 4 and 100 ng/ml bFGF media, respectively. The average number of colonies from triplicate wells was presented for each culture (n = 1). Error bars represent standard deviation. (c). Representative images of ALP stained cultures as described in (b). (d). Top row: MEF feeder cells demonstrated similar morphological changes in response to different bFGF concentrations as shown by hMSCs feeder cells in Figure 1; Middle row: representative H1-MEF colonies in 0, 04 and 4 ng/ml bFGF media 4 days post initial plating; Bottom row: representative H1-MEF colonies in 0, 04 and 4 ng/ml bFGF media 8 days post initial plating.

To compare how exogenous bFGF affects H1, H9 and DF19 colony growth on MEF feeder cells, H1-MEF-8 ng (P32), H9-MEF-8 ng (P27) and DF19-MEF-100 ng (P35) were each split evenly by collagenase into 0 vs. 8 ng/ml bFGF media or 0 vs. 100 ng/ml bFGF media, respectively, and continued to culture and split identically for 1 more passage before alkaline phosphatase (ALP) staining. For both H1-MEF and DF19-MEF, the lack of exogenous bFGF resulted in drastic reduction of total number of surviving colonies as well as significant increase in the percentage of colonies displaying clear differentiation characterized by enlarged flat cells with increased cytoplasma/nucleus ratio and reduced ALP staining intensity (Figure 8a and table 1), indicating that exogenous bFGF was required for promoting colony survival and preventing colony differentiation and in those two cell lines. For H9-MEF however, the effect appeared to be restricted to differentiation and not on total colony numbers during the 12-day culture (Figure 8a and table 1), indicating that H9-MEF is less dependent on exogenous bFGF compared to H1-MEF and DF19-MEF in terms of colony survival.

**Table 1:** H1, H9 and DF19 colony distribution in 0 vs. 8 or 0 vs. 100 ng/ml bFGF culture.

	H1		H9		DF19	
[bFGF] ng/ml	0	8	0	8	0	100
Undifferentiated	52	432	472	548	208	564
Differentiated	56	76	164	12	348	56
Total	108	508	636	560	556	620
**Differentiated/Total**	**51.8%**	**15.0%**	**25.8%**	**2.1%**	**62.6%**	**9.0%**

To also compare how exogenous bFGF affects pluripotent stem cell clonogenicity on MEF feeder cells, H1-MEF-8 ng (P32) and DF19-MEF-100 ng (P33) were dissociated into single cells by accutase and plated at 2×10^5^ cells/well into 0, 0.4 and 4 ng/ml bFGF media or 0, 4 and 100 ng/ml bFGF media, respectively, 3 wells of each. Culture was continued for 12 days before ALP staining and the number of total colonies and differentiated colonies in each well was counted. For both cell lines, the total number of colonies was not significantly affected by the concentration of exogenous bFGF, but the percentage of highly differentiated colonies was significantly increased as the concentration of bFGF was reduced (Figure 8b and 8c), indicating that exogenous bFGF was not required for single cell attachment or division but was required to suppress differentiation. Consistently, colonies also displayed differential morphological changes in response to different exogenous bFGF concentrations, starting 4 days after single cell plating. Compared to colonies in 4 ng/ml bFGF media, H1-MEF colonies in 0 or 0.4 ng/ml bFGF media had high proportion of cells containing shining vacuole-like structures (Figure 8d, middle row), which further deteriorated after prolonged culture, resulting in completely differentiated/degenerating colonies (Figure 8d, bottom row).

In summary, in contrary to cultures on hMSCs feeder cells, H1, H9 and DF19 cultures on MEF feeder cells require exogenous bFGF to inhibit differentiation in order to allow colony growth and expansion.

### Exogenous bFGF inhibits thawing efficiency in a dosage dependent manner across all three cell lines

**Figure 9:**
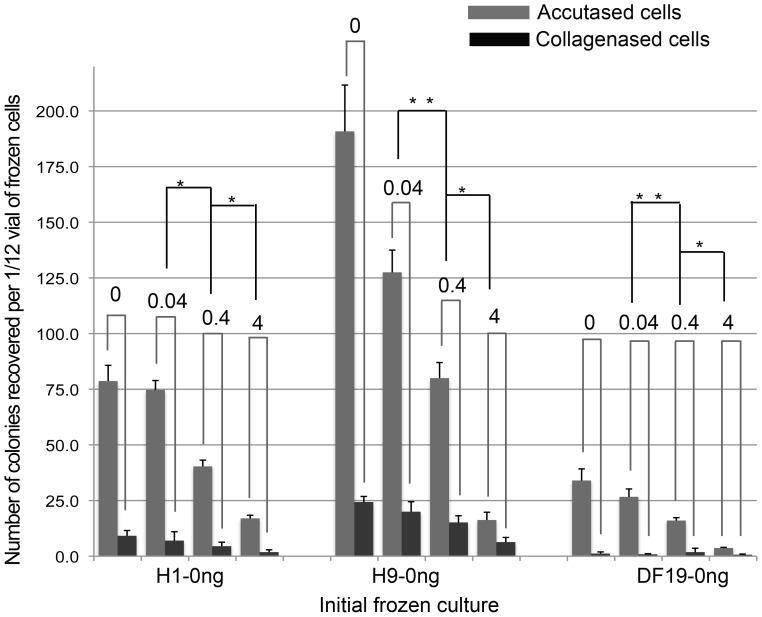
Exogenous bFGF inhibits thawing efficiency in a dosage dependent manner across all three-cell lines. Identical wells of H1-0 ng, H9-0 ng or DF19-0 ng culture were each frozen individually into a single vial after dissociation by collagenase or accutase. Subsequently each vial of frozen cells was plated equally into 0, 0.04, 0.4 or 4 ng/ml bFGF media, 3 wells of each. The average number of colonies from triplicate wells was presented for each culture (n = 2). Error bars represent standard deviation. Asterisks indicate statistical differences between different bFGF culture conditions in the accutased group. **: P<0.1; *: P<0.01. See also Figure S2.

Since exogenous bFGF supplement inhibited both colony growth (Figure 3) and clonogenicity rate across all three cell lines cultured on hMSCs feeder, we examined whether it could also inhibit thawing recovery from freezing. To test that, identical wells of H1-0 ng, H9-0 ng or DF19-0 ng culture were individually frozen down into single vials after cells were either dissociated by collagenase or accutase. One (for accutased cells) or two (for collagenased cells) frozen vials of each cell type was then thawed out and plated equally into 0, 0.04, 0.4 and 4 ng/ml bFGF media, 3 wells of each. Double amount of cells were plated for collagenased cells due to typical low thawing efficiency. Colonies were then ALP stained and counted after 12 days of culture. This allowed for parallel comparison between collagenased vs. accutased groups, as well as comparison among different bFGF culture conditions within each group. Consistently across all three cell lines, accutased cells recovered significantly better than collagenased cells (Figure 9 and Figure S2), indicating that cells frozen as single cells had greater capacity to survive freezing/thawing compared to cells frozen as clusters. In addition, bFGF demonstrated a dosage dependent inhibitory effect on freezing/thawing recovery across all cell lines, which was reflected by the statistically significant differences observed between different bFGF culture conditions within each accutased group, as well as an overall trend of decline in the number of colonies recovered from freezing/thawing in response to increased bFGF concentration in culture media within each collagenased group (Figure 9 and Figure S2). The relatively low thawing efficiency of DF19 compared to H1 and H9 were further confirmed by an independent repeat of the same experiment (data not shown).

### Exogenous bFGF at 20 or 100 ng/ml promoted DF19 differentiation in a dosage dependent manner but had limited effect on H9 cells

**Figure 10:**
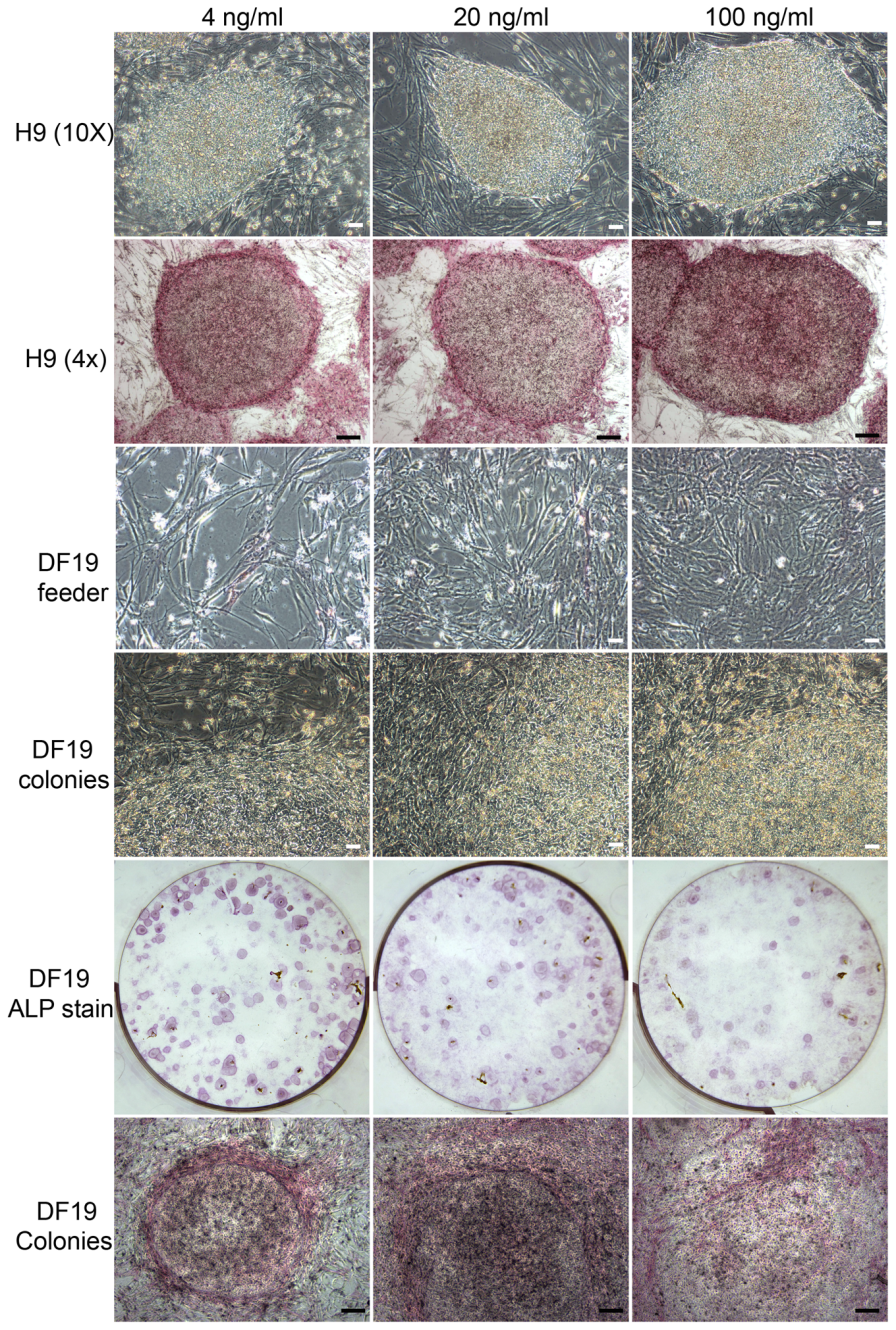
bFGF at 20 or 100/ml promoted DF19 differentiation in a dosage dependent manner but had limited effect on H9 cells. Top two rows: Bright-field images and ALP stained images of H9-4 ng, H9-20 ng and H9-100 ng cultured in parallel at P40+11; Middle two rows: Bright-field images of feeder cell layers and colonies in DF19-4 ng, DF19-20 ng and DF19-100 ng cultures at P33+6, with the latter demonstrating more blurred boundaries between feeder cells and colonies in DF19-20 ng and DF19-100 ng; Bottom two rows: ALP stained images of DF19-4 ng, DF19-20 ng and DF19-100 ng cultures at P33+6. Scales in white: 50 µM; Scales in black: 200 µM.

To examine how bFGF could affect hESCs/hiPSCs long-term culture at concentrations greater than 4 ng/ml, H9 (P40) and DF19 (P33) were each cultured in parallel in media supplemented with 4, 20 or 100 ng/ml bFGF on MtC-hMSCs feeder cells. H1 cells were not tested due to severe differentiation observed in 4 ng/ml bFGF culture. For H9 cells, all three concentrations of bFGF could sustain its long-term culture without noticeable differences in colony growth rate or ALP staining intensity (Figure 10, top two rows) and culture was discontinued at P40+13. For DF19 cells however, there was a gradual increase in differentiation in response to exogenous bFGF concentration in a dosage dependent manner and the severity increases over increased passages. Differentiated cells were predominantly fibroblast like cells, resulting in a denser feeder cell layer after each passage split, with the highest density in 100 ng/ml bFGF culture and the lowest in 4 ng/ml bFGF culture (Figure 10, middle two rows). ALP staining highlighted individual colonies with defined boundaries in the 4 ng/ml bFGF culture, whereas in the 20 or 100 ng/ml bFGF culture, boundaries between colonies and feeder cells were much more blurred (Figure 10, bottom two rows). Due to the gradual increase of differentiation occurring in the higher concentration bFGF cultures, culture was discontinued at P33+8.

### The effect of exogenous bFGF on H9 clonogenicity is similar regardless at 4 ng/ml or higher

**Figure 11:**
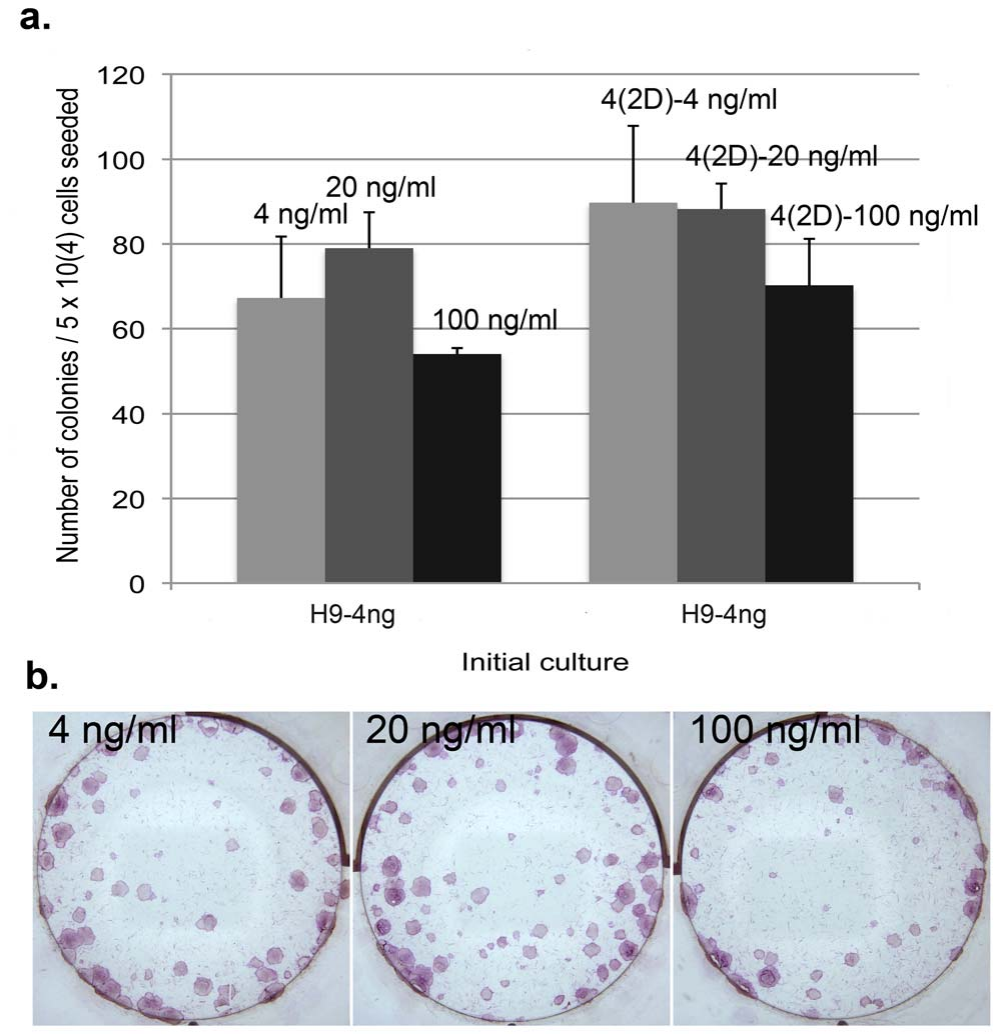
The effect of exogenous bFGF on H9 clonogenicity is similar at 4/ml or higher. (a). Initial culture of H9-4 ng at P40+10 was dissociated into single cells by accutase and plated into 4, 20 or 100 ng/ml bFGF media at 5×10^4^ cells/well, 2 wells of each, with or without 2 days of pre-incubation in 4 ng/ml bFGF media. Average number of colonies from duplicate wells was presented for each culture condition (n = 1). No statistically significant difference was observed between different culture conditions. (b). Representative ALP stained images of H9 culture in 4, 20 or 100 ng/ml bFGF media derived from H9-4 ng single cells.

Despite the similar growth rate observed among H9 cultures in 4 ng/ml or higher concentration bFGF, we decided to further probe potential differences by examining clonogenicity in those concentrations. Initial culture of H9-4 ng at P40+10 was dissociated into single cells by accutase and plated into 4, 20 or 100 ng/ml bFGF media at 5×10^4^ cells/well, 2 wells of each, with or without 2 days of pre-incubation in 4 ng/ml bFGF media. Culture was continued for 12 days before ALP staining and the number of total colonies was counted. In both scenarios, there was no significant difference observed between different culture conditions (Figure 11), indicating that the effect of bFGF on H9 clonogenicity reached plateau at 4 ng/ml and higher concentration did not exert additional effect.

### Pluripotency verification of H1, H9 and DF19 cultured in media supplemented with 0, 0.4, 4, 20 or 100 ng/ml bFGF by immunostaining, flow cytometry and RT-PCR

To characterize the pluripotency of H1, H9 and DF19 after long-term culture in media supplemented with 0–100 ng/ml of bFGF, immunostaining, flow cytometry and RT-PCR were applied to examine several pluripotency markers' expression.

**Figure 12:**
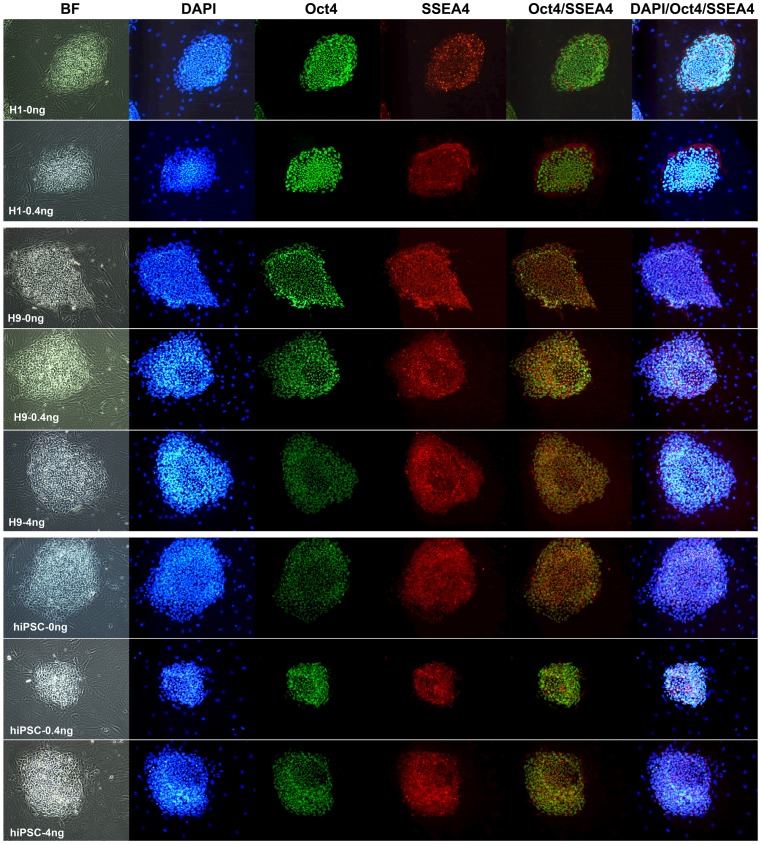
All cell lines cultured in 0, 0.4 or 4/ml bFGF media (excluding H1-4 ng) expressed both Oct 4 and SSEA4. BF, bright field; DAPI, a blue fluorescent nucleic acid stain; Oct4, a pluripotent nuclear marker labeled by green fluorescence; SSEA4, a pluripotent cell surface marker labeled by red fluorescence; Oct4/SSEA4, overlay of both markers; DAPI/Oct4/SSEA4, overlay of all 3 markers.

**Figure 13:**
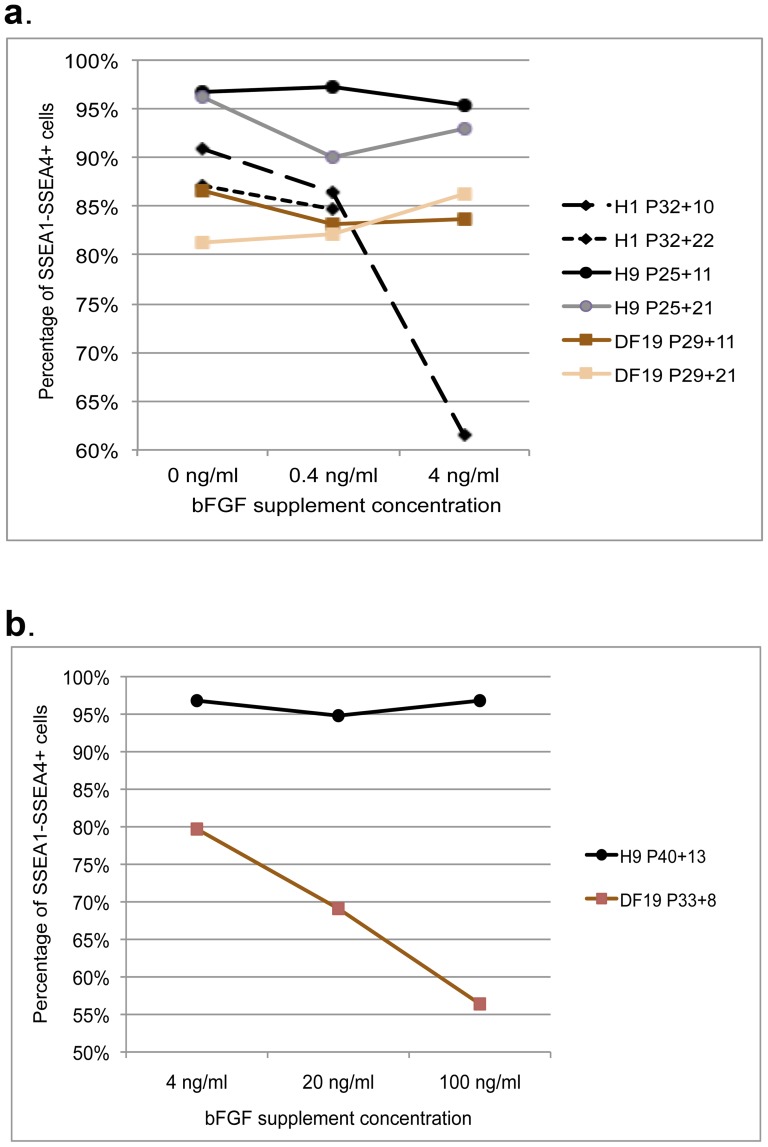
Flow cytometry analysis of SSEA1 and SSEA4 expression in all cell cultures. A. H1, H9 and DF19 cells were analyzed after both 10/11 and 21/22 passages of culture on Ad-hMSCs feeder cells in 0, 0.4 or 4 ng/ml bFGF media. B. H9 and DF19 cells were analyzed after 13 and 8 passages of culture respectively on Ad-hMSCs feeder cells in 4, 20 or 100 ng/ml bFGF media. See also Figure S3.

For H1, H9 and DF19 cultured in 0, 0.4 or 4 ng/ml bFGF media (excluding H1-4 ng) at P32+11, P25+11 and P29+11 respectively, cells were fixed and immunostained using antibodies against pluripotent markers Oct 4 and SSEA4. As expected, all cell lines demonstrated strong expression of both markers in colonized pluripotent stem cells but not in the feeder cells (Figure 12). This was further validated by flow cytometry analysis using antibodies against two cell surface markers, SSEA1, a differentiation marker, and SSEA4, a pluripotency marker, after 10 or 20 passages. Over 95% of H9 cultures, regardless of the bFGF concentrations, were SSEA4 positive and SSEA1 negative (SSEA4+/SSEA1−) at P25+11, which slightly dropped to 90–95% at P25+21 (Figure 13a and Figure S3). Similarly about 85–91% of H1 cells in 0 or 0.4 ng/ml bFGF media were SSEA4+/SSEA1− at both P32+10 and P32+22, whereas about 81–87% of DF19 cells in all concentrations of bFGF were SSEA4+/SSEA1− at both P29+11 and P29+21 (Figure 13a and Figure S3). H1-4 ng at P32+10 had severe differentiation morphologically and correspondingly only contained 61.5% of SSEA4+/SSEA1− cells (Figure 13a and Figure S3). Overall, there was no significant difference among different bFGF conditions within the same cell line, though the H9 cell line consistently had greater percentage of SSEA4+/SSEA1− cells as compared to H1 and DF19.

In addition, parallel cultures of both H9 and DF19 in 4, 20 or 100 ng/ml bFGF media were also examined for SSEA1 and SSEA4 expression. In all H9 cultures 95% or greater percentage of cells were SSEA4+/SSEA1− at P40+13, but only 56–80% of DF19 were SSEA4+/SSEA1− at P33+8 (Figure 13b and Figure S3). This was consistent with morphological observations made over long-term culture of these cells, in which bFGF demonstrated dosage dependent induction of differentiation in DF19 cultures, but had no dosage-dependent effect on H9 cultures (Figures 10 and 11).

**Figure 14:**
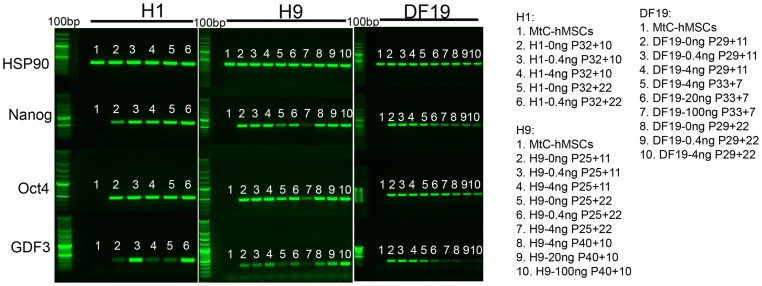
Expression of Oct4, Nanog and GDF3 was detected in all pluripotent cell cultures by RT-PCR. Expression of HSP90, a house keeping gene, was detected in all samples including mitomycin C treated hMSCs feeder cells (MtC-hMSCs). Expression of Nanog, Oct4 and GDF3 were restricted to all pluripotent cell samples and not expressed in MtC-hMSCs (H1 and H9, n = 3; DF19, n = 2). Expression of all genes was negative in water and RNA control sample lanes (not shown). See also Figure S4.

Finally, semi-quantitative RT-PCR was also carried out to examine additional pluripotent marker gene expression including Oct4, Nanog and GDF3, in an attempt to see if subtle differences in expression levels of these genes might be detected among cultures exposed to different bFGF concentrations. All 3 genes were detected in all pluripotent cell cultures but not in MtC-hMSCs feeder cells (Figure 14), as expected. It is noteworthy that GDF3 demonstrated higher expression level in 0.4 ng/ml cultures compared to 0 ng/ml cultures in both H1 and H9 cells, but lower in DF19 cells, after 22 passages of long-term parallel culture (Figure S4).

### Pluripotency verification of H1, H9 and DF19 cultured in media supplemented with 0, 0.4, 4, 20 or 100 ng/ml bFGF by embryoid body and teratoma formation

**Figure 15:**
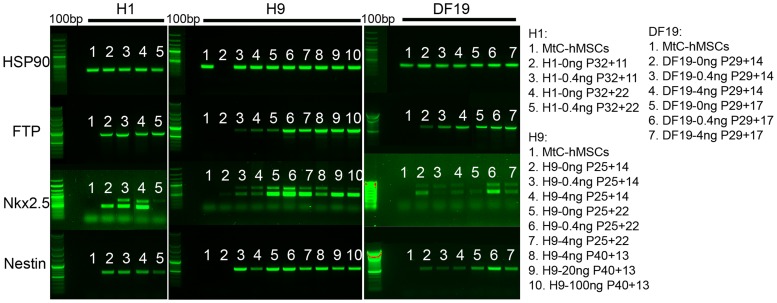
Expression of 3 germ lineage markers α-AFP, Nkx2.5 and Nestin was detected in EBs formed from all cell lines by RT-PCR. Expression of HSP90, a housekeeping gene, was detected in all samples except for H9-0 ng at P25+14. Expression of α-AFP, Nkx2.5 and Nestin were detected in all EBs formed from all three cell lines except for H9-0 ng at P25+14, but not expressed in MtC-hMSCs (n = 3). Expression of all genes was negative in water and RNA control sample lanes (not shown).

One of the major characteristics of pluripotent stem cells is their ability to differentiate into all cell types of the body. To examine the cells' ability to differentiate after being long-term cultured on hMSCs feeder cells in media supplemented with different concentrations of bFGF, embryoid bodies (EBs) were made from all three cell lines after they had been cultured in 0, 0.4, 4, 20 or 100 ng/ml bFGF media for 8–22 passages, excluding H1-4 ng, DF19-20 ng and DF19-100 ng, due to severe differentiation observed after about 7 passages. Expression analysis of 3 germ-layer specific genes, α-AFP (alpha-fetoprotein, endoderm), Nkx2.5 (cardiac marker, mesoderm) and Nestin (neuronal marker, ectoderm), were detected in all EBs formed from all three cell lines, except for H9-0 ng at P25+14 (Figure 15). The lack of germ-layer specific gene expression in H9-0 ng at P25+14 was most likely due to technical errors, as the housekeeping gene HSP90 was missing in that sample as well, and a later passage of H9-0 ng at P25+22 demonstrated expression of all 3 genes examined. In addition, beating cardiomyocytes were observed in H1-0 ng-derived EBs at both P32+15 and P32+22, and in EBs derived from H9-0 ng, H9-0.4 ng and H9-4 ng at P25+22. No beating cardiomyocytes were observed in H1-0.4 ng-derived EBs nor in any DF19-derived EBs, which appeared to correlate with the relatively weak expression of Nkx2.5 in these samples as well (Figure 15).

**Figure 16:**
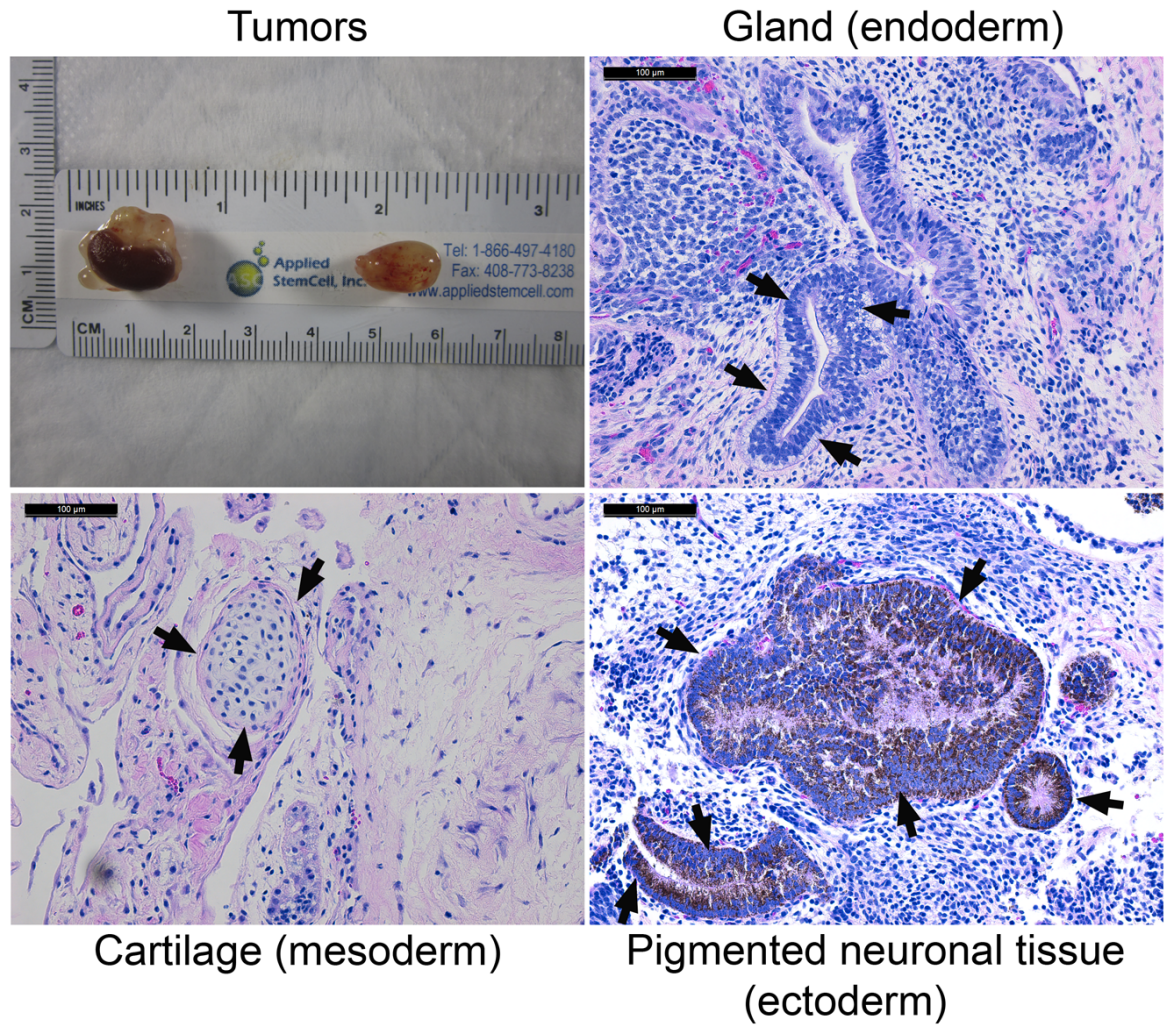
H1-0 ng at P32+19 gave rise to teratoma that contained all three germ layer lineage tissues. Tumor: one kidney tumor (left) and one testis tumor (right) collected on day 19 post injection. Variously differentiated tissues from isolated tumors that represent the three germ layers are shown and indicated by arrows. Scale: 100 µM.

To further validate the cells' ability to differentiate into all 3 germ layer lineages after long-term culture in the absence of exogenous bFGF supplement, H1-0 ng at P32+19 was chosen for teratoma formation test. Around 1.5–2 million cells/site were injected into kidney or testis of 3 SCID mice and tumors were harvested on day 39 post-injection from both organs of all 3 mice (Figure 16). Histological section and staining identified tissues representing all 3 germ layers, endoderm, mesoderm and ectoderm (Figure 16). This combined with other data presented above clearly indicated that the H1-0 ng cells were still pluripotent after long-term culture on hMSCs feeder cells even in the absence of exogenous bFGF.

### Long-term cultured H1-0 ng and H9-0 ng cells had normal karyotype, whereas both DF19-0 ng and DF19-4 ng cells had abnormal karyotype characterized by trisomy 12

**Figure 17:**
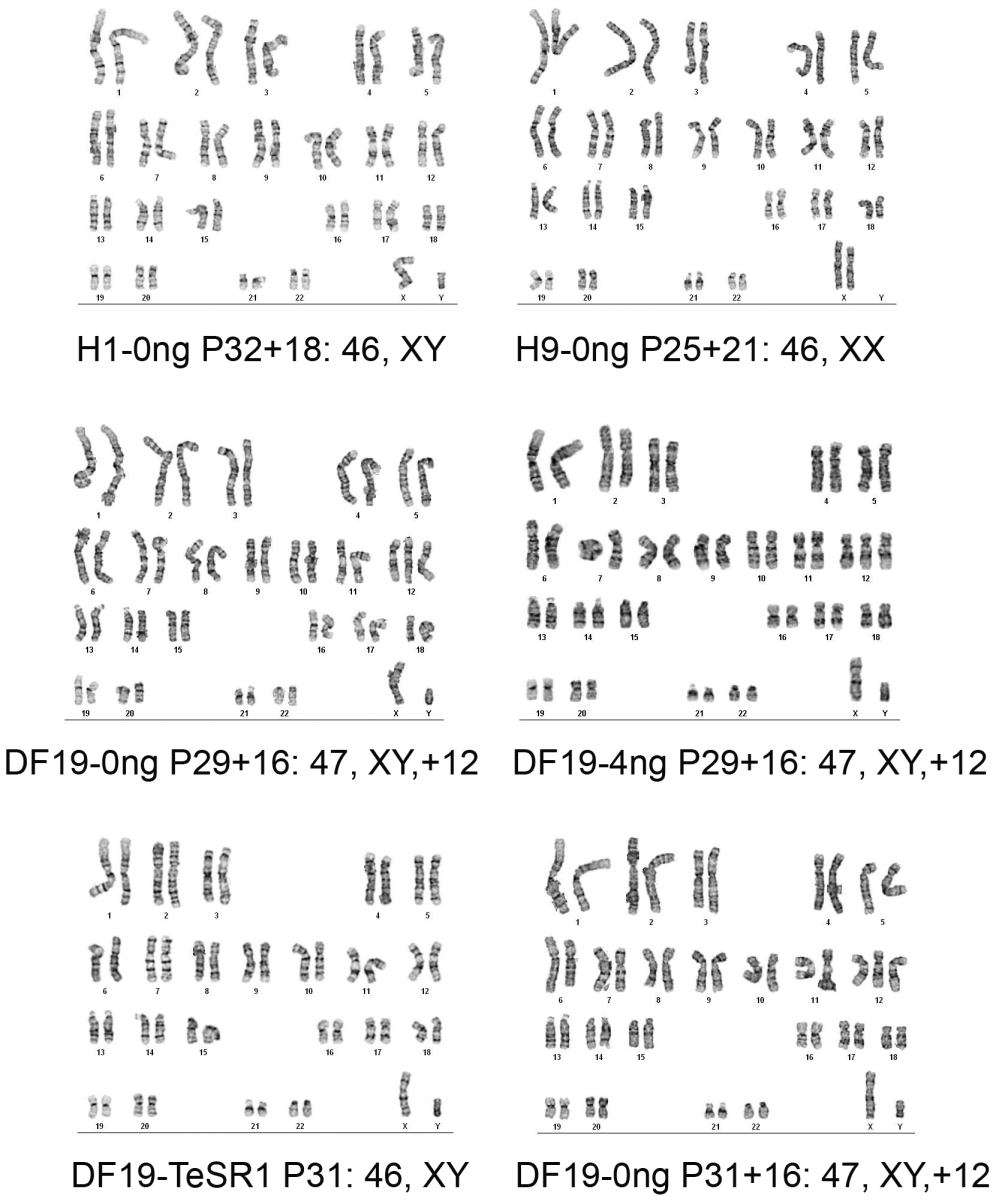
Long-term cultured H1-0 ng and H9-0 ng cells had normal karyotype, whereas both DF19-0 ng and DF19-4 ng cells had abnormal karyotype characterized by trisomy 12. Clonal aberration was observed in all cells examined in both DF19-0 ng and DF19-4 ng cultures at P29+16. Similar aberration was also observed in an independent culture of DF19-0 ng at P31+16, whereas the original culture of DF19 in TeSR1 media at P31 had normal karyotype.

Since standard culture of H1, H9 and DF19 has always been supplemented with exogenous bFGF at 4 ng/ml or higher, we examined whether long-term culture of these cells in the absence of bFGF could still maintain normal chromosomal integrity. G-band characterization was carried out for H1-0 ng at P32+18 and H9-0 ng at P25+21, both of which demonstrated normal karyotype at the resolution of 425−275 (Figure 17, top row). However, for DF19-0 ng at P29+16, clonal aberration with trisomy 12 was observed in all cells examined (Figure 17, middle row). To see whether this was unique to DF10-0 ng culture, DF19-4 ng culture at P29+16 was also examined. Surprisingly, it also demonstrated identical abnormal karyotype (Figure 17, middle row). To rule out the possibility that the abnormality observed in DF19-0 ng and DF19-4 ng was a random event, an independent culture of DF19-0 ng originated from DF19 in TeSR1 media at P31 was also carried out and examined at P31+16, and again it showed the same abnormality in all cells examined. As a control, the original culture of DF19 in TeSR1 at P31 from which the DF19-0 ng and DF19-4 ng were derived had normal karyotype, indicating that the clonal aberration observed in DF19-0 ng and DF19-4 ng cells was acquired during their long-term culture on hMSCs feeder cells, which appeared to be independent of the concentrations of exogenous bFGF.

### H1, H9 and DF19 demonstrated differential growth capacity in MtC-hMSCs conditioned media with or without bFGF supplement

**Figure 18:**
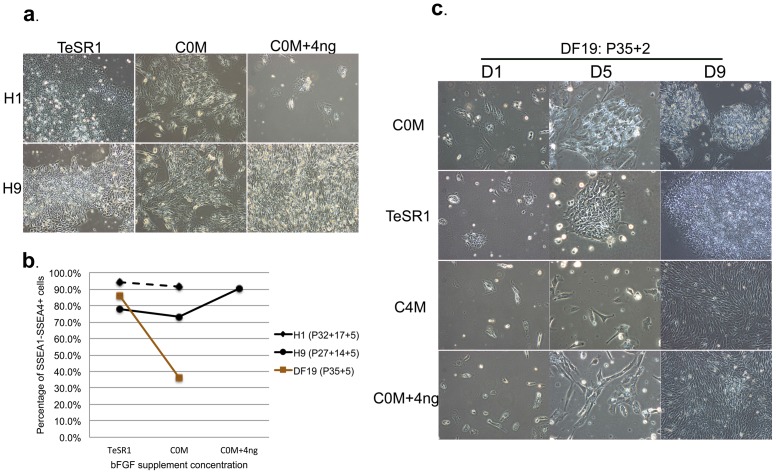
H1, H9 and DF19 demonstrated differential growth capacity in Ad-hMSCs conditioned media with or without bFGF supplement. A. Images of H1 (P32+17+2) and H9 (P25+14+2) cultured in TeSR1, C0M (hESCs/hiPSCs media conditioned with MtC-hMSCs in the absence of bFGF) or (C0M+4 ng) (hESCs/hiPSCs media conditioned with MtC-hMSCs in the absence of bFGF and subsequently supplemented with 4 ng/ml bFGF). B. Flow cytometry analysis of SSEA1−SSEA4+ cells in H1 (P32+17+5), H9 (P25+14+5) and DF19 (P35+5) after 36–38 days of culture in TeSR1, C0M or (C0M+4 ng). C. Images of DF19 (P35+2) cultured in TeSR1, C0M, (C0M+4 ng) or C4M (hESCs/hiPSCs media conditioned with MtC-hMSCs in the presence of 4 ng/ml bFGF) on D1, D5 and D9 after plating. See also Figure S5.

It was shown that two hESCs lines, H9 and AND1, could be long-term sustained in conditioned media by human mensenchymal stem cells in the absence of exogenous bFGF [45]. Due to differences observed among different cell lines as well as within the same cell line when cultured on hMSCs feeder cells with or without bFGF supplement, we compared H1, H9 and DF19 culture in MtC-hMSCs conditioned media with or without bFGF supplement. H1-0 ng and H9-0 ng cells at P32+17 and P25+14, respectively, were accutased and plated as single cells on matrigel at 3×10^5^ cells/well in TeSR1, hESCs/hiPSCs media conditioned with MtC-hMSCs in the absence of bFGF for 1 day (C0M) or C0M supplemented with 4 ng/ml bFGF (C0M+4 ng). Media was changed on a daily basis and cells were passaged at the same density subsequently. At P32+17+2, few H1 cells cultured in (C0M+4 ng) survived and the culture was discontinued (Figure 18a). The remaining cultures were continued for additional 3 passages for a total of 36 days of culture (H1 P32+17+5 & H9 P25+14+5) before they were subjected to flow cytometry analysis. Both H1 in C0M and H9 in (C0M+4 ng) demonstrated over 90% SSEA1−/SSEA4+ cells, while H9 in C0M demonstrated about 73.3% of SSEA1−/SSEA4+ cells similar to those in TeSR1 (77.9%) (Figure 18b and Figure S5), indicating that MtC-hMSCs conditioned media could sustain short-term culture of both cell lines and maintain pluripotent stem cell marker SSEA4 expression but each cell line demonstrated distinct tolerance for exogenous bFGF supplement in such culture.

For DF19 cells, to avoid using cells with karyotypic abnormality, the original cells cultured in TeSR1 at P35 were accutased and plated as single cells on matrigel at 1×10^5^ cells/well in TeSR1, C0M or (C0M+4 ng). To also probe whether exogenous bFGF at 4 ng/ml could inhibit the secretion of growth factors important for pluripotent stem cell growth, an additional culture in hESCs/hiPSCs media conditioned with MtC-hMSCs in the presence of 4 ng/ml bFGF for 1 day (C4M) was included as well. Media was changed on a daily basis and cells were passaged at the same density. At P35+2, the cells began to show distinct morphologies on day 5 (D5) after plating, and by day 9 (D9), cells in C4M and (C0M+4 ng) all differentiated into fibroblast-like cells, while cells in C0M maintained colony shape (Figure 18c). Both C0M and TeSR1 cultures were continued until P35+5 for a total of 38 days of culture before they were subjected to flow cytometry analysis, accumulating 36.2% and 86% SSEA1−/SSEA4+ cells, respectively (Figure 18b and Figure S5). This indicated that unlike H1 or H9 cells, MtC-hMSCs conditioned media was not capable of sustaining short-term culture of DF19, with or without bFGF supplement.

## Summary

In summary, we present evidences that bFGF supplement exerts dosage and cell line dependent effect by examining parallel cultures of two hESCs lines (H1 and H9) and one hiPSCs line (DF19-9-7T) in media supplemented with 0, 0.4 or 4 ng/ml of bFGF for up to 23 passages, as well as parallel cultures of H9 and DF19 in media supplemented with 4, 20 or 100 ng/ml bFGF for up to 13 passages on hMSCs feeder cells. We demonstrated: I) Exogenous bFGF supplement inhibited growth expansion, clonogenicity and freezing/thawing efficiency in a dosage dependent manner across all three cell lines tested; II) bFGF exerted differential effects on different cell lines. It induced acute differentiation of H1 after 7 passages at 4 ng/ml, and inhibited DF19 culture expansion in a dosage dependent manner at 4, 20 and 100 ng/ml, while permitting long-term culture of H9 at the same concentrations with limited dosage effect. Similar differential effects were observed when the three cell lines were cultured in hMSCs-conditioned media with or without bFGF supplement, and again H9 demonstrated the best tolerance in the presence of exogenous bFGF; III) Stem cell pluripotency was maintained for all cell lines cultured on hMSCs feeder cells in 0, 0.4 or 4 ng/ml bFGF media (excluding H1-4 ng) as well as H9 cultured in 4, 20 and 100 ng/ml bFGF media by verifying the expression of pluripotent marker genes as well as the expression of three germ-layer lineage marker genes in embryoid bodies. Teratoma formation was also confirmed for H1-0 ng culture; and lastly, IV) Different cell lines harbor distinct chromosomal stability in the same culture condition, as karyotypic abnormality characterized by trisomy 12 was observed in DF19-0 ng and DF19-4 ng cultures but not in H1 or H9.

## Discussion

In the past decade or so, many different culture conditions have been developed that could support the pluripotency of hESCs and hiPSCs. However, the molecular mechanisms underlying the regulation of pluripotency are still unclear. More specifically, even though exogenous bFGF has been used almost universally as a growth factor in supporting hESCs/hiPSCs growth, its mode of action is still illusive. MEF cells do not express bFGF [44], [46]. When cultured on MEF feeder cells, hESCs were found to express endogenous bFGF at the level around 0.1 ng/ml [44], and knockdown of the intracrine bFGF induced differentiation of hESCs [1]. It was proposed that intrinsic and exogenous bFGF worked synergistically to maintain the undifferentiated growth and survival of hESCs, with exogenous bFGF also promoting hESCs attachment [1]. Our study with H1, H9 and DF19 demonstrated that passage of colonies in small clusters using collagenase in the absence of bFGF resulted in drastic reduction in colony numbers (H1 and DF19) as well as significantly increased differentiation (H1, H9 and DF10) during a short period of time (Figure 8a and Table 1). However, single cell colonization assay of all three cell lines in the presence or absence of bFGF demonstrated that exogenous bFGF did not significantly affect single cell attachment, but promoted differentiation (Figures 8b–d). It was likely that reduced colony numbers observed in short-term passage with collagenase in 0 ng/ml bFGF media was a result of the inability of differentiated cells to re-colonize. Our combined results implied that exogenous bFGF was indispensable for preventing differentiation of hESCs cultured on MEF, but not on colony attachment.

In contrast to MEFs, human feeder cells including HFF, HPF and human bone marrow derived stromal cells could support hESCs culture in the absence of exogenous bFGF, as demonstrated in a previous study [43], [44]. In this study, we further expand such feeder cell types to include human adipose-derived mesenchymal stem cells. Unlike MEFs, human feeder cells such as HFF and HPF do secret bFGF [44], [46], and it was estimated that HFF secreted bFGF at the levels ranging from 8 to 44 pg/ml [46]. In a separate study, it was shown that Ad-hMSCs secreted more than three fold higher levels of bFGF than HFFs (8.5 vs. 2.7 pg/ml) [45]. In addition, the amount of bFGF production by H1 on MEF or HPF might also be significantly different from each other [44], suggesting that the differential capacity of MEF and human feeder cells to secret bFGF could be a potential factor determining their differential dependence on exogenous bFGF to support hESCs/hiPSCs culture. Interestingly, our study for the first time revealed that in all three cell lines tested, H1, H9 and DF19, exogenous bFGF within the range of 0–4 ng/ml demonstrated dosage dependent inhibitory effect on the cell's ability to attach and survive on hMSCs, whereas bFGF had a dosage dependent inhibitory effect on the differentiation of these cells on MEFs. The two distinct effects exert opposing outcome in the stem cells' self-renewal capacity, indicating that the same concentration of exogenous bFGF might carry distinct modes of action within the two different microenvironments provided by MEF vs. hMSCs.

Indeed, besides bFGF, differences in the production of additional growth factors between MEF and human feeder cells as well as between different human feeder cell types have also been identified. Both MEF and HFF cells have been found to express comparable levels of TGF-β1 that were further induced by exogenous bFGF [46]. However, MEF cells express much higher level of activin A than HFF [46]. In addition, hESCs-MEF secreted IGF-II that was shown critical for sustaining self-renewal and pluripotency of hESCs [2], but production of IGF-II varied considerably among different human feeder cells [47]. HFF but not hMSCs secreted detectable basal level of IGF-II regardless of the presence or absence of hESCs, but both feeder cells types were able to support hESCs (HS181 and SHEF1) growth in conditioned media (CM) produced in the presence of 8 ng/ml bFGF [47], indicating IGF-II might be dispensable for hESC pluripotency. Interestingly only hMSCs but not HFF conditioned media produced in the absence of bFGF could support long-term culture of hESCs (H9 and AND1) [47]. The ability of different feeder media to support different pluripotent cell lines might indicate that individual pluripotent cell line has its own requirement for self-renewal. In this study with H1, H9 and DF19 cultured in different concentrations of bFGF supplement, we revealed that 4 ng/ml bFGF media could support long-term culture of H9, but induced acute differentiation of H1. Similarly, hMSCs conditioned media in the presence of 4 ng/ml bFGF could support H9 but not H1 long-term culture, and neither hESCs/hiPSCs media conditioned in the absence of bFGF nor the presence of 4 ng/ml bFGF could maintain the pluripotency of DF19. In addition, bFGF at 20 or 100 ng/ml could successfully sustain H9 long-term culture, but induced severe differentiation in DF19. Our results suggested that each feeder cell type would create a unique microenvironment depending on the culture media composition and this microenvironment might support the culture of one hESCs/hiPSCs line, but not another, due to the distinct culture requirement of each pluripotent stem cell line.

Along the same line of discovery, our study revealed that DF19 was karyotypically unstable when cultured on hMSCs feeder, regardless of exogenous bFGF concentration, whereas both H1 and H9 appeared stable, further indicating distinct culture condition requirements for successfully maintaining different pluripotent stem cell lines. Coincident with our findings, it was shown that different hESCs cultured in the same media conditions demonstrated different karyotypic instability, which was attributed to each cell line's inherent property [48]. One potential explanation for the exclusive development of abnormal karyotype in DF19 is that unlike H1 and H9, DF19 was derived in the presence of SV40 Large T gene along with six reprogramming genes (*Oct4*, *Sox2*, *Nanog*, *Lin28*, *Klf4* and *c-Myc*) cloned in an episomal vector [31]. While the transgenes were subsequently lost, it is possible that their presence resulted in inherent chromosomal instability when the subsequent culture condition was significantly deviated from the original. It remained to be seen whether the same holds true to other hiPSCs derived in similar fashion. Another potential explanation was that unlike H1 and H9, DF19 was derived and maintained in media with 100 ng/ml bFGF supplement [31], and subsequently switched to TeSR1 media which also contains 50 ng/ml bFGF. Switching the cells from such high concentrations of bFGF to 0 ng/ml bFGF media might have driven the selection of cells that had acquired mutation and gained growth advantage during the adaptation process. In support of this latter theory, our study with single cell colonization assay clearly indicated that pre-adaptation to a specific bFGF concentration helped enhance the survival rate of all three cell lines in the same condition. A drastic change in bFGF concentration might have posted a highly stringent condition for the DF19 cells to survive and hence increased the chance of mutation to adapt.

Finally, our study also revealed that all three cell lines tested had significant advantage over growth, clonogenicity and thawing recovery when cultured on hMSCs feeder in the absence of bFGF supplement as compared in the presence of bFGF. It is noteworthy that such advantage in clonogenicity could potentially significantly facilitate the derivation of new hiPSCs lines on hMSCs feeder cells, as traditional hiPSCs derivation was all carried out in the presence of high concentrations of bFGF [49].

In summary, our study demonstrated a dosage and cell line dependent inhibitory effect of exogenous bFGF on human pluripotent stem cells cultured on inactivated hMSCs, highlighting the importance of understanding the intricate molecular network regulating stem cell self-renewal and the potential need of developing individually tailored culture condition for each individual hESCs/hiPSCs cell line.

## Materials and Methods

### hMSCs culture and Mitomycin C treatment

Ad-hMSCs were purchased from Fisher Scientific (SV3010201) and cultured in Hyclone Advance STEM expansion media (Fisher Scientific, SH30875KT). Passage 5–6 hMSCs cells were used in all experiments described. Multipotency of these cells was validated by independent osteogenic and adipogenic differentiation (data not shown) [50]. For mitomycin C treatment, confluent cells on 10-cm plates were treated with 10 µg/ml mitomycin C (cat# M4287, Sigma) for about 2.5 to 3 hours and washed with PBS 3 times before being used or frozen.

### H1, H9 and DF19 culture and dissociation

H1(WA01), H9 (WA09) and DF19 (iPS DF19-9-7T) were all purchased from the National Stem Cell Bank at www.wicell.org. H1 and H9 were initially expanded in hESCs media composed of 80% DMEM/F12 (cat#11330057, Life Technologies), 20% knockout serum replacer (cat#10828028, Life Technologies), 1% NEAA (cat# 11140050, Life Technologies), 4 ng/ml bFGF (cat# 100-18B, PeproTech), 1 uM L-Glutamine (G8540, Sigma) and 0.07 µM beta-mercaptoethanol (ES-007-E, Millipore) on MEF feeder cells (GSC-6001M, mitomycin inactivated CF-1 cells, GlobalStem), whereas DF19 was expanded in TeSR1 media (5850, Stem Cell Technologies) on matrigel (354234, BD). For culturing on inactivated hMSCs, all cell lines were grown in hESCs media supplemented with different concentrations of bFGF. Cells were long-term passaged using 1 mg/ml collagenase (cat# 17104-019, Life Technologies) and colonies were detached by physical scraping using the tip of a glass pipet after 7 minutes of collagenase incubation. Single cell dissociation was carried out using accutase (cat# A1110501, Life Technologies) by following the provided manufacturer's protocol. Cells were passed through a sterile cell strainer (cat# 08-771-01, Thermofisher Scientific) to achieve single cell homogeneity and counted using the Countess automated cell counter (cat# C10227, Life Technologies), average of 3 chambers/sample.

### Alkaline Phosphatase Staining

ALP staining was done with Sigma Alkaline Phosphatase staining kit Ref 86R-1KT per manufacturer's protocol.

### Immunostaining

Human iPSC or ESCs were cultured on 6-well plates with glass coverslips. On day 5, media was removed and cells were rinsed with PBS then fixed with 4% paraformaldehyde for 30 min at room temperature (RT). Cells were then washed twice with 0.1% Tween 20 in PBS (PBT) at RT. For OCT-4 primary antibody, cells were permealized with 1% Triton-X 100/PBS for 30 minutes at RT. After blocking with 4% Goat Serum in PBS for 30 minutes at RT, cells were incubated with primary antibodies, rabbit Oct-3/4 antibodies (sc-9081, Santa Cruz) and mouse SSEA-4 (MAB4304, Millipore) at 1∶200 dilution in PBS, in a humidity chamber overnight at 4°C, followed by washing with PBT 3 times at RT, 5 minutes each, and subsequent incubation with conjugated 2° antibodies, Alexa Fluor-488 nm affinity pure donkey anti-rabbit and Alexa Fluor-594 nm affinity pure Donkey anti-mouse (Jackson Immunoresearch), at 1∶300 dilution in PBS for 1 hour at RT. Cells were then washed with PBT 3 times at RT, 5 minutes each and counterstained with 1 µg/mL DAPI in PBS for 5 minutes.

### Flow Cytometry

The following procedures were followed for the preparation of flow cytometry samples: The day before staining and fixing, coat FACs tubes with 1% BSA overnight in 4C. Thaw out 1 vial of MtC-hMSCs onto one 10-CM plate for negative control. Reagents and Media: 1. Prepare 100 mL of wash buffer (WB) by adding 2 mL of Fetal Calf Serum to 98 mL PBS. Store at 4C for up to one month. 2. Prepare fixation buffer (FB) by diluting 4% Paraformaldehyde to 2% with PBS. Make fresh each use, keep on ice and discard at the end of the day. 3. Neutralization Media: Make MEF Media (90% DMEM-F12 and 10% FBS) for trypsin or use 0 ng/mL Media for accutase. Cell Preparation: 1. Aspirate media and wash 2X with 3 mL of PBS. 2. Add 1 ml Accutase to each well and Incubate at 37C for 9 min. 3. Use a Pasteur pipet and manually dislodge all cells and transfer to 15 mL conical tube. 4. Add 4 ml Neutralization Medium (MEF Media or 0 ng/mL media) to inactivate accutase. Note: Add some media to wash off any leftover cells on the wells. 5. Centrifuge at 1000 rpm for 5 minutes. 6. Resuspend cell pellet in 3 mL per well with 1X WB. 7. Add the cells to the center of the cell strainer and allow cells to pass through the filter by gravity. 8. Rinse the cell strainer with 2 mL WB. 9. Pipet out 1 ml and use that to count cells. 10. Centrifuge again for 5 minutes at 1000 rpm. 11. Resuspend cell pellet at 5×10^6^ cells/mL in Wash Buffer. Note: The minimum number of cells required is 3×10∧6 cells/mL. For MtC-hMSC feeder cells: Trypsinize cells with 0.05% trypsin and resuspend cells in MEF Media. Count cells. At least 7×10^5^ cells are required. Resuspend this in 220 uL Wash Buffer and add 100 uL each to tubes 6 and 7. Staining and Fixation: 1. Label FACs tubes 1–7. (12×75 mm round bottom test tubes). 2. Add 100 uL of the cell suspension to tubes 2–5. For tubes 6–7, add 100 uL each of the Mtc-hMSCs suspsension (see above note). 3. Add the rest of the hESC/hiPSC cell suspension to tube 1. 4. Add antibodies according to the chart below. 5. Incubate for 30 min at 4C in the dark. Wrap tubes in foil. Occasionally flick the tube to resuspend the cells. 6. Add 2 mL WB to each tube and centrifuge for 10 minutes at 1000 rpm. 7. Decant the supernatant and resuspend the cell pellet in 100 uL of FB (Fixation Buffer). 8. Vortex and incubate cells at RT for 30 minutes in the dark. 9. Add 2 mL of PBS and centrifuge for 10 min at 1300 rpm. 10. Decant supernatant and resuspend the cells in 300 uL of WB. 11. Store in 4–8C for up to one week before FACS analysis. Sample Labeling: Tube 1: hESCs/hiPSCs, no antibodies; Tube 2: hESCs/hiPSCs, isotypes IgG3-488 nm and IgM-647 nm; Tube 3: hESCs/hiPSCs, anti-SSEA4-488 nm and IgM-647 nm; Tube 4: hESCs/hiPSCs, anti-SSEA1-647 nm and IgM-488 nm; Tube 5: hESCs/hiPSCs, anti-SSEA4-488 nm and anti-SSEA1-647 nm; Tube 6: MtC-hMSCs, isotypes IgG3-488 nm and IgM-647 nm; and Tube 7: MtC-hMSCs, anti-SSEA4-488 nm and anti-SSEA1-647 nm.

Flow cytometry data was acquired through a CyAn ADP-9color equipment (Beckman Coulter) at the City of Hope flow cytometry core and analyzed using the FlowJo software by Tree Star Inc.

### Embryoid body (EB) formation

hESCs or hIPSC colonies were cultured on 6 well plates until 100% confluent and were detached with 3 mg/mL dispase (17105-041, Invitrogen) for 20 minutes at 37C until most colonies were entirely lifted. Remaining attached colonies were manually dislodged by gently tapping on the plates. Cells were then transferred to a 50 ml conical tube where they were allowed to settle by gravity. The supernatant was carefully removed and washed 3X with 10 ml of DMEM high glucose (4.5 g/ml glucose) basal media. Cell aggregates were re-suspended in freshly made EB media consisted of 90% DMEM-high glucose, 10% FBS (hMSC-defined, Cat# SH30070.03, Hyclone), 1 uM glutamine (Cat# G8540, Sigma), 0.07 uM β-ME (Cat# ES-007E, Millipore), and 1% NEAA (Cat# 11140050, Life Technologies). EBs were plated onto 3 wells of a 6-well ultralow attachment plate and maintained in the EB media for 6 days followed by serum starvation for 24 hr by replacing regular EB media with a media substitute that contained no FBS. EBs were subsequently attached on gelatin-coated wells and maintained in regular EB media for additional 14–17 days, with media change every 2–3 days.

### Teratoma formation

Fox Chase SCID-beige, male, 6-week-old mice (vendor, Charles River) were used for H1 cell injection. A total of 3 mice were injected in both the kidney capsule and testis loci with 1.5–2 million cells/site and tumors were harvested from both injection sites in each mouse 39 days post injection. Tumor tissues were fixed with 10% formalin overnight, embedded in paraffin and sectioned at 5-µM thickness for H&E staining. The Applied StemCell, Inc. provided this service.

### Gene expression by reverse transcription (RT)-PCR analysis

Total RNA was isolated from cells with the RNeasy kit (QIAGEN 74104). SUPERSCRIPT II reverse transcriptase (Invitrogen 11752050) was used for RT. PCR was carried out using the HotStarTaq plus master mix kit (cat# 203645, Qiagen) or the Fast cycling PCR kit (cat# 203741, Qiagen). Primer sequences and PCR conditions are summarized in supplement table I.

### Mycoplasma Testing

All cell cultures were tested for Mycoplasma contamination using ATCC Universal Detecting kit 30-1012K per manufacturer's protocol.

### Statistical analysis

Unpaired student *t*-test was used to evaluate the statistical differences between two treatment groups.

## Supporting Information

Figure S1 **H1, H9 and DF19 were contaminant free after long-term cultur.** Late passage cultures of H1-0 ng, H9-0 ng and DF19-0 ng were tested for the presence of mycoplasma contaminants using the Universal Mycoplasma Detection Kit by ATCC, which detects over 60 species of *Mycoplasma*, *Acholeplasma*, *Spiroplasma* and *Ureaplasma*.
(TIF)

Figure S2 **Exogenous bFGF inhibits thawing efficiency in a dosage dependent manner across all three-cell lines.** Identical wells of H1-0 ng, H9-0 ng or DF19-0 ng culture were individually frozen down into single vials after cells were either dissociated by collagenase or accutase. One (for accutased cells) or two (for collagenased cells) vials of each frozen cell type was then thawed out and plated equally into 0, 0.04, 0.4 and 4 ng/ml bFGF media, 3 wells of each. Colonies were then ALP stained and counted after 12 days of culture. Representative image of each culture was presented.
(TIF)

Figure S3 **Summaries of flow cytometry histograms.** H1, H9 and DF19 cultured in media supplemented with 0, 0.4, 4, 20 or 100 ng/ml bFGF were co-stained with antibodies against SSEA1 (Alexa647, y-axis) and SSEA4 (Alexa 488, x-axis). The same expression thresholds were applied in each set of cultures.
(TIF)

Figure S4 **GDF3 demonstrated differential expression levels in different culture conditions after long-term culture.** Expression of GDF3 in 0.4 or 4 ng/ml culture was quantified relative to that in 0 ng/ml culture and normalized by the expression of housekeeping gene *Hsp90*. Data shown are the mean value of repeats (H1 and H9, n = 3; DF19, n = 2). Error bars represent standard deviation.
(TIF)

Figure S5 **Summaries of flow cytometry histograms.** H1, H9 and DF19 cultured in TeSR1, C0M (MtC-hMSCs conditioned media in the absence of bFGF) or (C0M+4 ng) (MtC-hMSCs conditioned media in the absence of bFGF and subsequently supplemented with 4 ng/ml bFGF) for 36–38 days were co-stained with antibodies against SSEA1 (Alexa647, y-axis) and SSEA4 (Alexa 488, x-axis). The same expression thresholds were applied in all cultures.
(TIF)

Table S1 **Primer sequences and PCR conditions.** This table enlists the primer sequences and PCR conditions used for gene expression examination by RT-PCR.
(DOCX)
