# Supplementary figures and images for: Dosage and Cell Line Dependent Inhibitory Effect of bFGF Supplement in Human Pluripotent Stem Cell Culture on Inactivated Human Mesenchymal Stem Cells
Source: PLoS One. 2014 Jan 17;9(1):e86031. doi: 10.1371/journal.pone.0086031 (PMC3895015; doi:10.1371/journal.pone.0086031)

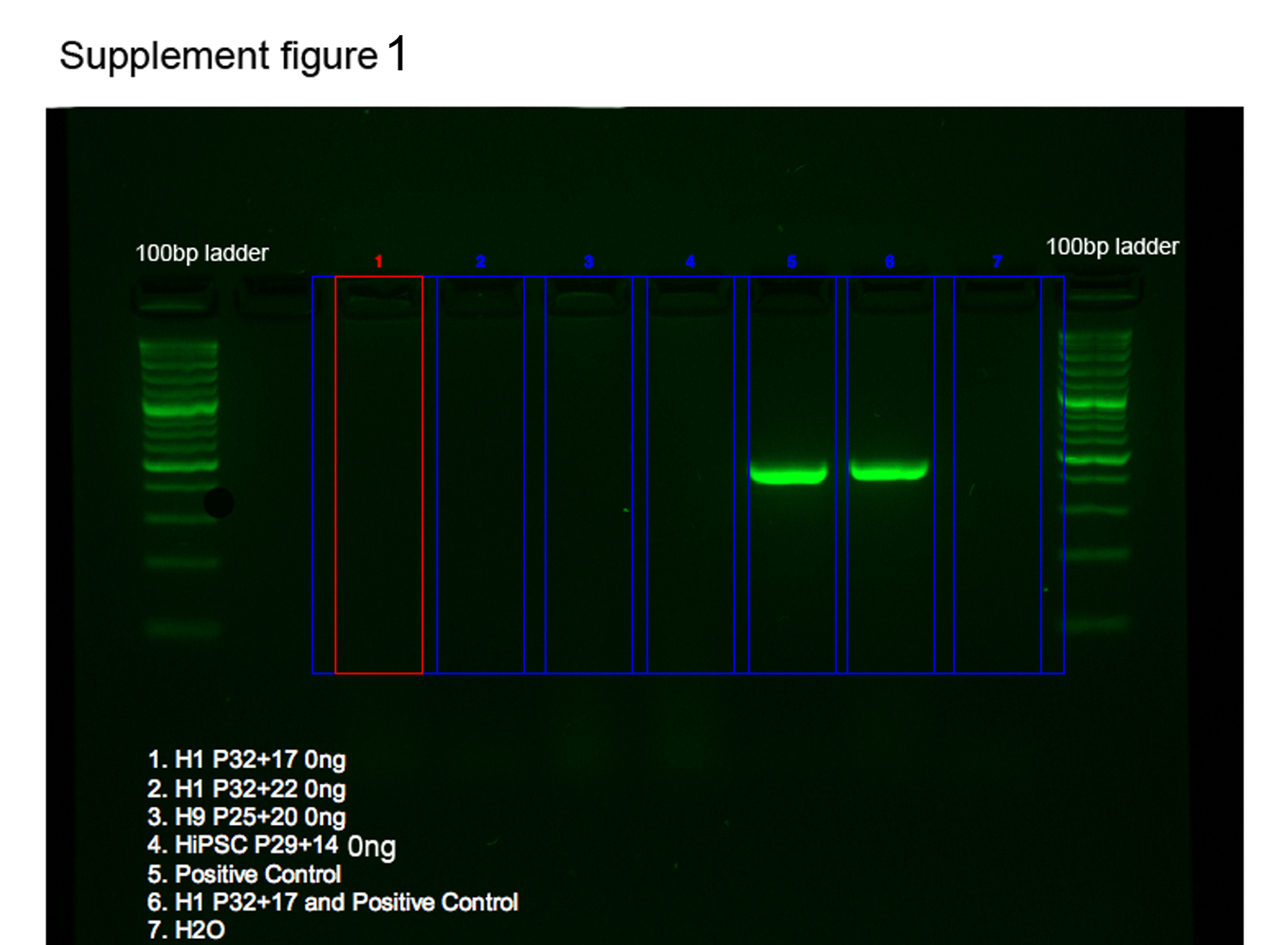

Supplement: Figure S1 — H1, H9 and DF19 were contaminant free after long-term cultur. Late passage cultures of H1-0 ng, H9-0 ng and DF19-0 ng were tested for the presence of mycoplasma contaminants using the Universal Mycoplasma Detection Kit by ATCC, which detects over 60 species of Mycoplasma, Acholeplasma, Spiroplasma and Ureaplasma. (TIF) [file pone.0086031.s001.tif]

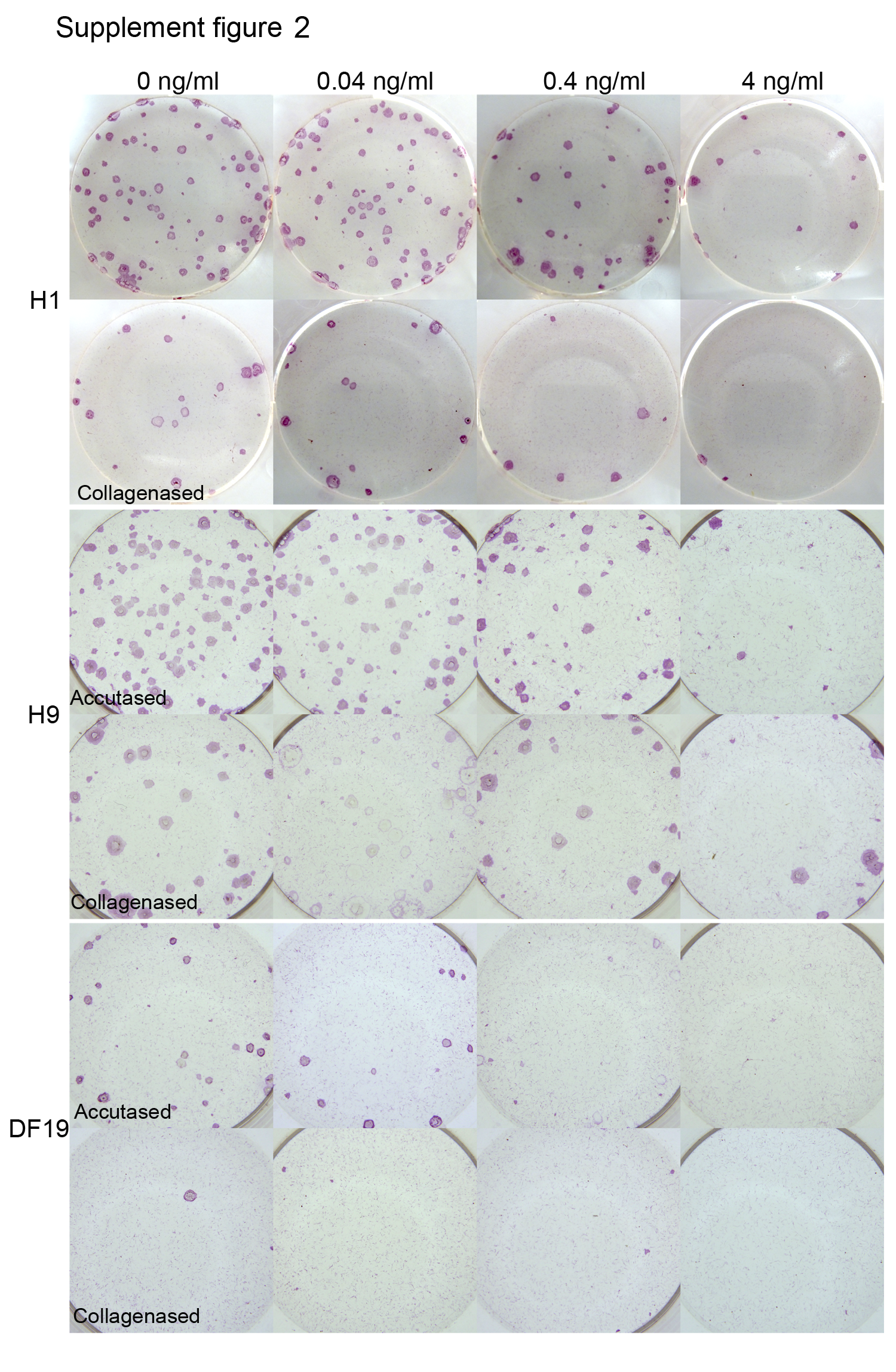

Supplement: Figure S2 — Exogenous bFGF inhibits thawing efficiency in a dosage dependent manner across all three-cell lines. Identical wells of H1-0 ng, H9-0 ng or DF19-0 ng culture were individually frozen down into single vials after cells were either dissociated by collagenase or accutase. One (for accutased cells) or two (for collagenased cells) vials of each frozen cell type was then thawed out and plated equally into 0, 0.04, 0.4 and 4 ng/ml bFGF media, 3 wells of each. Colonies were then ALP stained and counted after 12 days of culture. Representative image of each culture was presented. (TIF) [file pone.0086031.s002.tif]

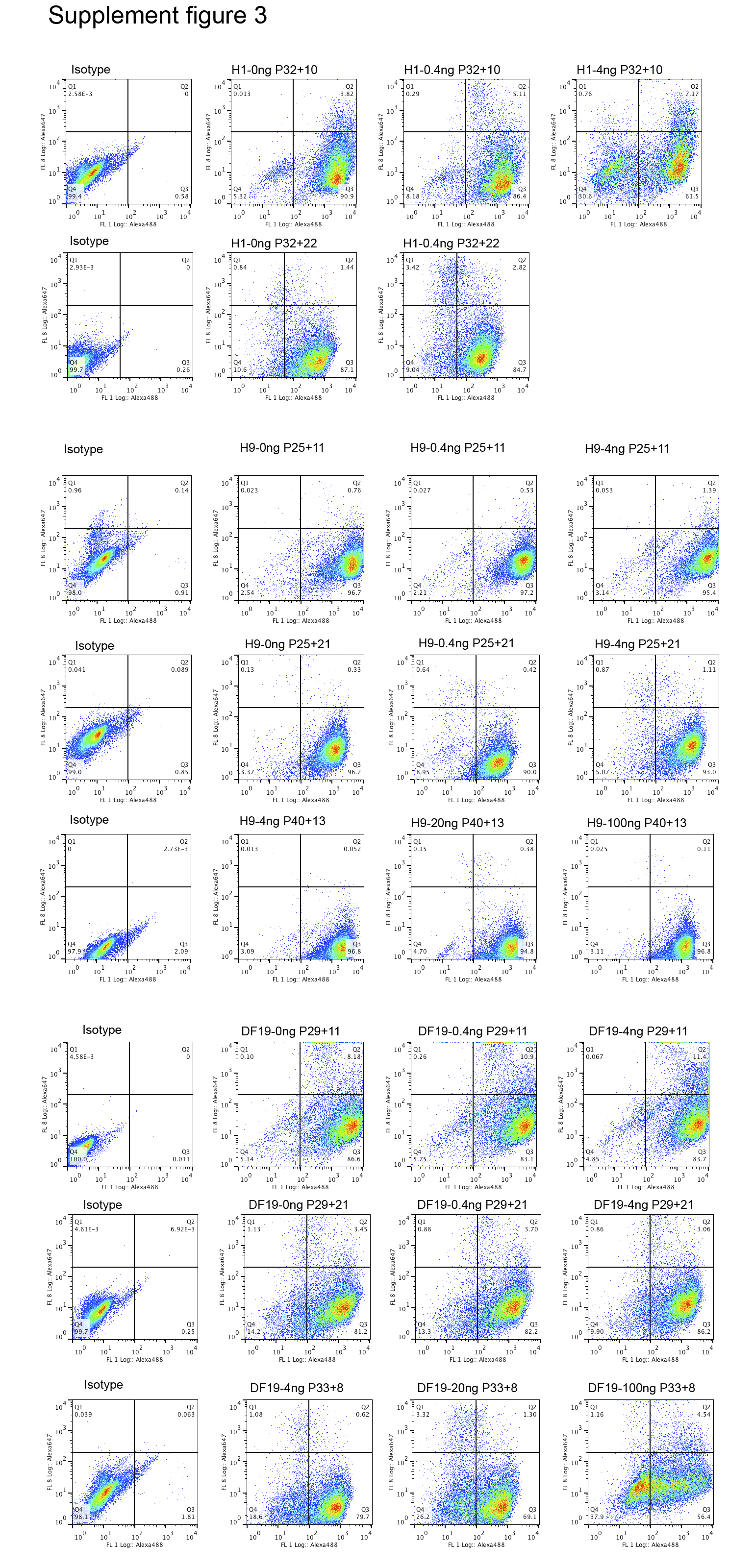

Supplement: Figure S3 — Summaries of flow cytometry histograms. H1, H9 and DF19 cultured in media supplemented with 0, 0.4, 4, 20 or 100 ng/ml bFGF were co-stained with antibodies against SSEA1 (Alexa647, y-axis) and SSEA4 (Alexa 488, x-axis). The same expression thresholds were applied in each set of cultures. (TIF) [file pone.0086031.s003.tif]

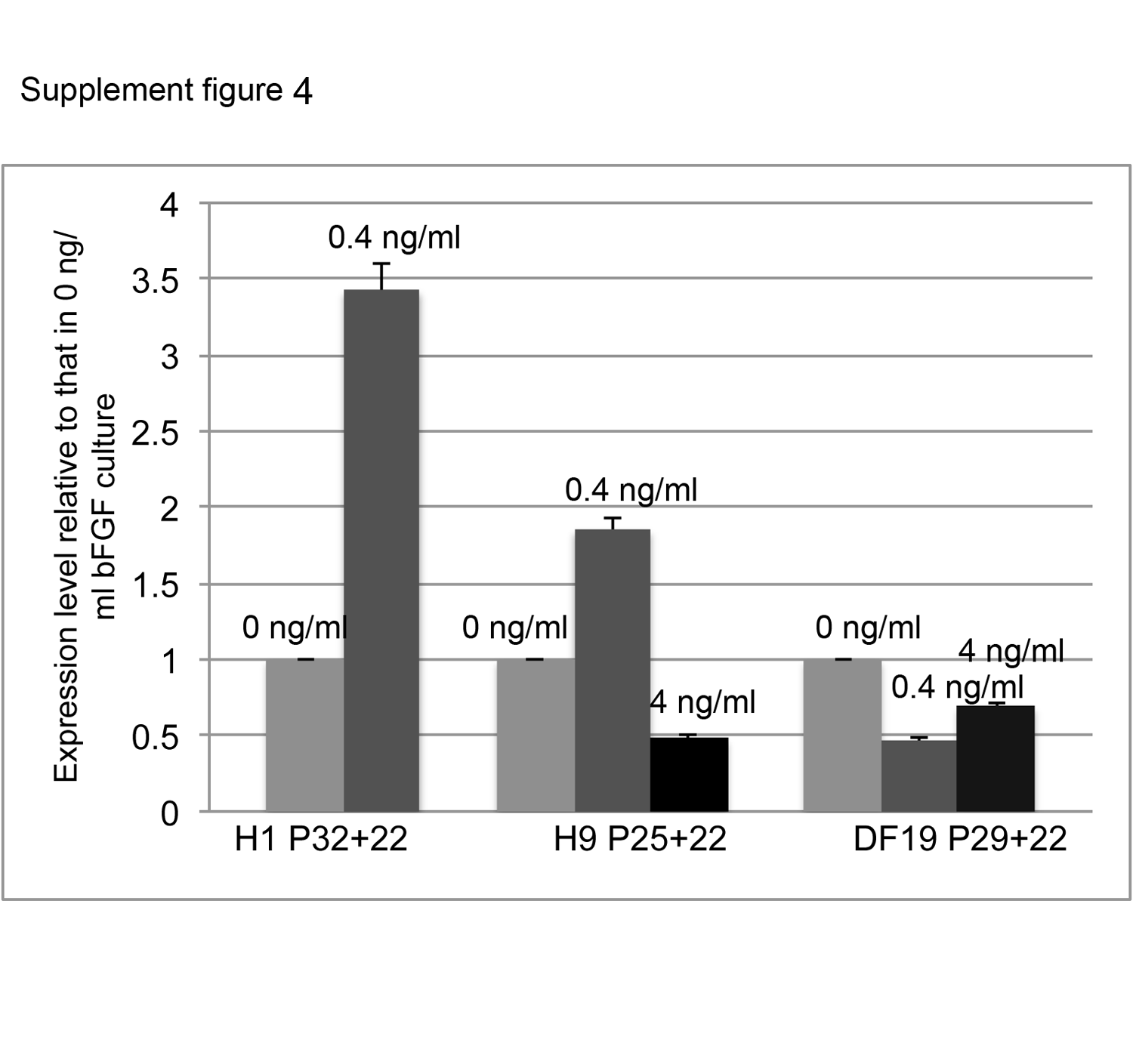

Supplement: Figure S4 — GDF3 demonstrated differential expression levels in different culture conditions after long-term culture. Expression of GDF3 in 0.4 or 4 ng/ml culture was quantified relative to that in 0 ng/ml culture and normalized by the expression of housekeeping gene Hsp90. Data shown are the mean value of repeats (H1 and H9, n = 3; DF19, n = 2). Error bars represent standard deviation. (TIF) [file pone.0086031.s004.tif]

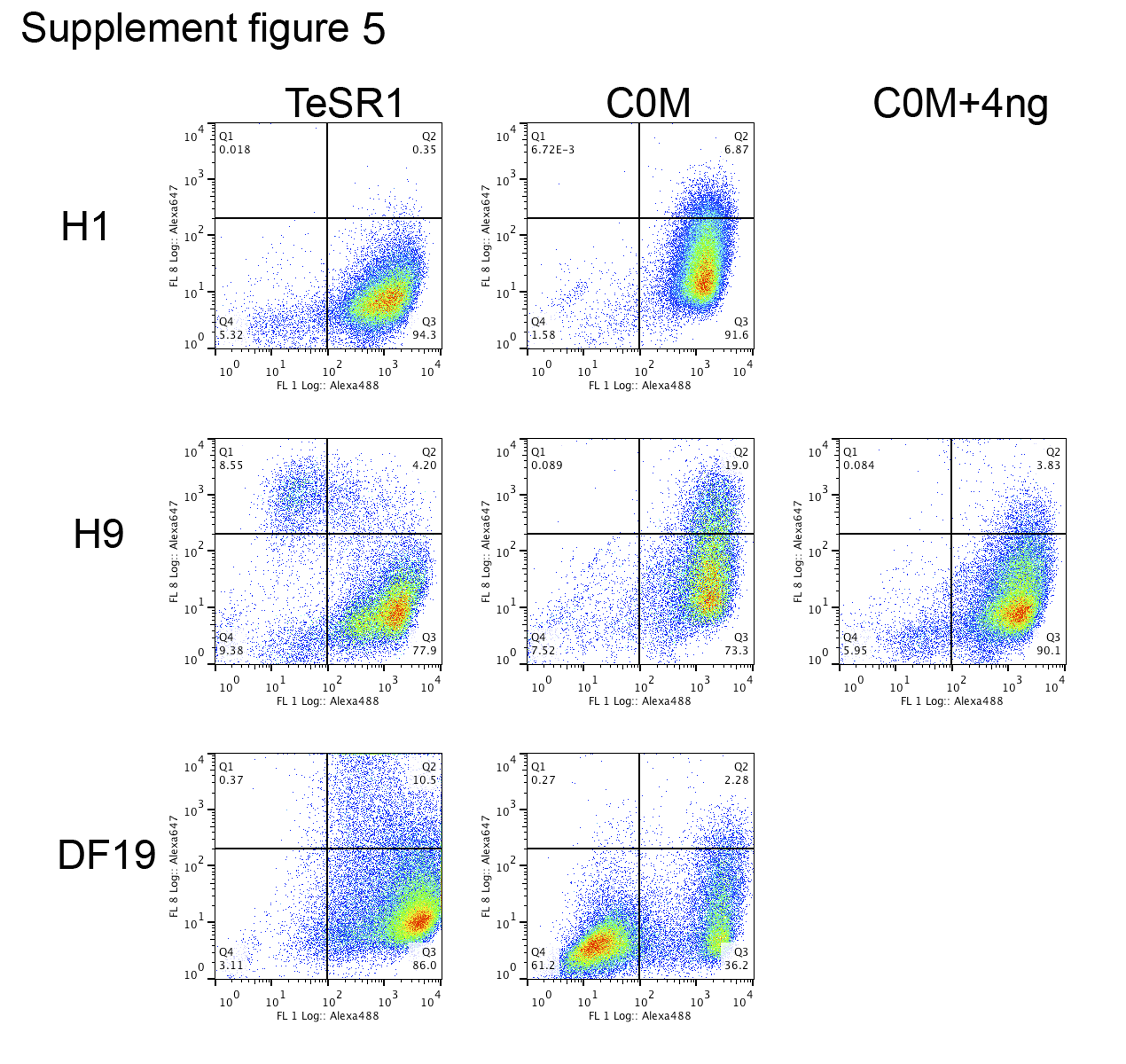

Supplement: Figure S5 — Summaries of flow cytometry histograms. H1, H9 and DF19 cultured in TeSR1, C0M (MtC-hMSCs conditioned media in the absence of bFGF) or (C0M+4 ng) (MtC-hMSCs conditioned media in the absence of bFGF and subsequently supplemented with 4 ng/ml bFGF) for 36–38 days were co-stained with antibodies against SSEA1 (Alexa647, y-axis) and SSEA4 (Alexa 488, x-axis). The same expression thresholds were applied in all cultures. (TIF) [file pone.0086031.s005.tif]
