# Supplementary material for: Dosage and Cell Line Dependent Inhibitory Effect of bFGF Supplement in Human Pluripotent Stem Cell Culture on Inactivated Human Mesenchymal Stem Cells
Source: PLoS One. 2014 Jan 17;9(1):e86031. doi: 10.1371/journal.pone.0086031 (PMC3895015; doi:10.1371/journal.pone.0086031)
Supplement: Table S1 — Primer sequences and PCR conditions. This table enlists the primer sequences and PCR conditions used for gene expression examination by RT-PCR. (DOCX) [file pone.0086031.s006.docx]

| **Gene name (hs.)** | **Forward primer 5'-3'** | **Reverse Primer 5'-3'** | **Product size (bps)** | **PCR condition**  **(Reg. Taq)** |
| --- | --- | --- | --- | --- |
| HSP90-beta | TACTTGGTGGCAGAGAAAGT | CTCATCTGAACCCACATCTT | 363 | 90C 30'', 60C 1', 72C 1' (30X) |
| GDF3 | CTG GGG TCT CCC GAG ACT TA | GGA CTG ACC GCA ACA CAA AC | 327 | 94C 30min,  57C 1min,  72C 1min (30X) |
| OCT4 | CGTCACCCCTGGTGCCGTGA | TGGCTGATCTGCTGCAGTGTGG | 438 | 94C 30min,  60C 1min,  72C 1min (30X) |
| Nanog | AGGAAGACAAGGTCCCGGTCA | GGCCTTCCCCAGCAGCTTCC | 561 | 94C 30min,  60C 1min,  72C 1min (30X) |
| NKX2.5 | GGACCCTAGAGCCGAAAAGAA | GATAGGCGGGGTAGGCGTTA | 458 (variant 1)  713/750 (variants 3/2) | 96C 5sec,  55C 5sec,  68C 15sec (FastTaq, 35X) |
| FTP | TTC TTT GGG CTG CTC GCT AT | CGC CAC AGG CCA ATA GTT TG | 865 | 94C 30min,  57C 1min,  72C 1min (30X) |
| Nestin | GAT CGC TCA GGT CCT GGA AG | AAC ACT CTA GAC CCA CCG GA | 639 | 94C 30min,  57C 1min,  72C 1min (30X) |

**Table S1**
